# Collective self-justification and resistance to integration: behavioral and social mechanisms within the Israeli ultra-orthodox community

**DOI:** 10.3389/fsoc.2026.1756939

**Published:** 2026-07-13

**Authors:** Guy Hochman

**Affiliations:** Baruch Ivcher School of Psychology, Reichman University, Herzliya, Israel

**Keywords:** decision-making, groups, Haredi, Homobiasos, motivated reasoning, polarization, religion, belongingness

## Abstract

**Background:**

Israel’s ultra-Orthodox (Haredi) community combines strong religious and cultural separation from secular society with increasing dependence on state institutions, labor markets, higher education pathways, and public resources. This tension is especially salient in debates over employment, secular curricula, and military service, where practical pressures for integration may conflict with communal authority, symbolic boundaries, and fears of secularization. The Haredi case provides a theoretically informative setting for examining the Homobiasos framework, which proposes that moral reasoning can function as a coherence-preserving system under identity threat.

**Methods:**

A cross-sectional national survey of 429 Haredi adults in Israel examined attitudes toward employment, education, military and civic service, communal belonging, national identity, and political representation across four Haredi subgroups: Lithuanian, Hasidic, Sephardic, and Modern-Others. Quantitative analyses were complemented by thematic analysis of substantive open-ended responses.

**Results:**

The findings revealed a patterned form of bounded adaptation. Openness was greater in domains that could be framed as pragmatic, economically necessary, or compatible with communal continuity, particularly in employment and women’s education. Resistance was stronger in domains perceived as threatening core symbolic boundaries: 53.3% opposed secular curricula in Haredi schools, and roughly two-thirds opposed an adapted military-service track. Qualitative narratives showed how adaptation was morally reframed as continuity, while nonparticipation was often justified as Torah study, communal protection, or alternative civic contribution.

**Conclusion:**

These findings suggest that collective moral reasoning may preserve group coherence by absorbing some forms of change while resisting others experienced as assimilation or moral defeat. Thus, integration in polarized societies may depend not only on incentives or factual correction, but on whether new obligations can become morally inhabitable within the group’s existing self-understanding.

## 1 Introduction

### 1.1 Moral reasoning as coherence regulation

Group conflicts are often described as disagreements over facts, interests, or policy (Dahrendorf, 1959; Simmel, 1955; Sherif, 1966, 1967; Tajfel and Turner, 1979, 1986). Yet many of the most persistent conflicts endure even when information is widely available, the practical stakes are clear, and the costs of non-cooperation are collectively recognized (Kahan, 2016; Olson, 1965; Ostrom and Walker, 1996; Taber and Lodge, 2006). In such cases, resistance to change may reflect not only ignorance, misperception, or strategic self-interest, but also the need to preserve moral coherence: the experienced fit among beliefs, values, identity, emotions, social belonging, and collective self-understanding (Hochman, 2026).

A large body of research on motivated reasoning, social identity, attribution bias, and moral cognition shows that people do not process socially consequential information as detached observers. Rather, they interpret evidence in ways that protect valued identities, moral commitments, and group loyalties (Ditto et al., 2019; Haidt, 2001; Jost and Banaji, 1994; Kahan, 2013; Kunda, 1990; Pettigrew, 1979; Tajfel and Turner, 1986). Individuals often explain their own group’s behavior in situational or morally justified terms while attributing out-group behavior to stable dispositions or moral deficiencies (Pettigrew, 1979; Hewstone, 1990). These processes can sustain collective confidence and belonging, but they can also reinforce polarization by making one’s own position feel not merely preferable, but morally necessary.

The Homobiasos framework extends this tradition by proposing that many forms of biased reasoning are not merely failures of rationality but regulatory responses to threats to coherence (Hochman, 2026). Under this view, reasoning is not always organized around accuracy alone. People may be committed to truth, evidence, morality, and rationality, yet still favor interpretations that allow them to remain morally, socially, and narratively intelligible to themselves and their group (Barkan et al., 2015; Cushman, 2020; Mazar et al., 2008). When evidence or social pressure threatens this coherence, reasoning may be recruited to reinterpret information, reframe obligations, shift responsibility, or reaffirm moral worth. Bias, in such cases, may function less as a random error and more as a tool for preserving a livable moral self.

Importantly, Homobiasos does not reject accuracy-based accounts of judgment or limitation-based accounts of bias. Many systematic errors clearly result from limited information and attention, weak numeracy, noisy inference, or reliance on heuristic thinking (De Neys, 2023; Evans and Stanovich, 2013; Hochman, 2024; Kahneman, 2011; Tversky and Kahneman, 1974). Instead, it explains why more information, deliberation, or corrective pressure may not reduce bias (Nyhan, 2021; Walter et al., 2019; Wittenberg and Berinsky, 2020). This is because the central problem is not cognitive difficulty but psychological cost. When accurate recognition threatens identity, dignity, moral standing, or group belonging, individuals and groups may preserve coherence even at epistemic, material, or social cost.

### 1.2 From individual coherence to collective self-justification

Although Homobiasos was developed primarily as a model of individual reasoning, its logic extends naturally to collective life when coherence is understood not only as a property of individual cognition but also as a socially sustained relation among shared values, public narratives, institutional norms, and recognized forms of belonging. I define collective coherence as the perceived compatibility between a group’s moral self-understanding, its normative boundaries, and its actual patterns of behavior, dependence, and adaptation. A community is collectively coherent when its members can interpret what the group does (e.g., compromises, refusals, dependencies, and changes) as consistent with what the group believes itself to be.

Collective coherence is not produced by a single group mind or by perfect consensus among members. Rather, it emerges through repeated and distributed processes of meaning-making: shared narratives, institutional routines, educational systems, leadership cues, symbolic boundaries, public discourse, religious or ideological reasoning, and recurring patterns of justification (Boltanski and Thévenot, 2006; Lamont and Molnár, 2002; Tole, 1993; Zilber, 2024). These processes provide common interpretive frames through which members learn which actions count as loyalty, necessity, threat, compromise, or betrayal. Because these frames are socially reproduced, they can preserve a shared sense of continuity even when individuals differ in practice, subgroup location, or private ambivalence.

The collective extension of Homobiasos concerns the social regulation of meaning under tension. Groups, like individuals, face contradictions between ideals and behavior, moral narratives and practical constraints, continuity and adaptation (Dillon, 1999; Ellemers et al., 2019; Öhman and Östman, 2007). These tensions are especially visible in communities whose shared identity rests on strong moral boundaries. In such communities, social changes are evaluated not only in instrumental terms, but also by whether they can be reconciled with the group’s values, authority structures, and symbolic self-understanding (Carnes and Lickel, 2018; Ellemers, 2018; Ellemers and Bos, 2012). This is the point at which coherence regulation becomes collective self-justification.

Collective self-justification refers to the social production of narratives, symbols, and moral explanations that preserve a group’s sense of virtue and continuity under conditions of tension. Such justification does not necessarily eliminate contradiction. Rather, it transforms contradiction into meaning (Halevy et al., 2025; Hochman et al., 2021). Dependence can be reframed as contribution, separation as protection, adaptation as necessity, and resistance as faithfulness. Through public discourse, religious or ideological reasoning, or shared interpretive frames, communities may absorb social change without experiencing it as a loss of identity (Alexander et al., 2004; Gongaware, 2011; Obradović and Howarth, 2018; Thumma, 1991; Ybema, 2010).

This dynamic can be described as limited modernization: behavioral or pragmatic adaptation without proportional ideological or normative transformation. A group may become more economically integrated, technologically exposed, or civically entangled while continuing to reject the broader cultural meanings associated with integration (Campbell, 2010; Eisenstadt, 2000; Neriya-Ben Shahar, 2017; Wetmore, 2007). According to Homobiasos, this is not necessarily incoherent but a stable regulatory solution. Practical adaptation can be tolerated when it can be framed as continuity, necessity, or service to the group; it is resisted when it appears to require moral assimilation, symbolic surrender, or betrayal of the collective self (Danbold and Huo, 2022; Hornsey and Hogg, 2000; Venus et al., 2019).

This approach also helps explain why polarization is often resistant to factual correction (Kahan, 2013, 2016; Nasie et al., 2014; Taber and Lodge, 2006). Groups in conflict frequently mirror one another’s defensive reasoning while perceiving only the other side as biased (Pronin et al., 2002, 2004, 2007; Silverstein and Flamenbaum, 1989). What appears externally as hypocrisy or irrationality may function internally as coherence regulation (Cusimano and Lombrozo, 2021; Festinger, 1957; Gawronski, 2012). The persistence of contradiction, therefore, is not always a breakdown of reasoning. It may reflect an effort to maintain moral order under the strain of social transformation (Gawronski, 2012).

### 1.3 The Haredi community as a case of bounded adaptation

Israel’s ultra-Orthodox, or Haredi, community provides a particularly informative context for examining collective coherence under pressure. From the outside, Haredi society is often perceived as a unified and insulated group. In practice, however, it is internally diverse and sociologically stratified. The community includes Lithuanian, Hasidic, Sephardic/Mizrahi, Modern-Haredi, and other hybrid or subgroup identities, each with distinct traditions, leadership structures, educational institutions, and patterns of engagement with wider Israeli society (Cahaner and Malchi, 2022; Caplan and Leon, 2024; Friedman, 1991; Leon, 2016, 2020, 2023). Recent research has documented increasing heterogeneity in employment, education, political attitudes, digital exposure, and civic engagement, especially among younger cohorts and women (Feldman, 2019; Finkelman, 2011; Malach and Cahaner, 2023; Perelman et al., 2024).

This internal diversity makes the Haredi case sociologically important. It cautions against treating the community as a single moral actor, while also raising a theoretically central question: how does a diverse and changing community preserve a shared sense of ideological continuity? The answer suggested by the Homobiasos framework lies not in the absence of contradiction but in regulating it. Haredi society can adapt in some domains while preserving strong resistance in others because domains differ in their symbolic meaning, perceived moral threat, and implications for communal authority.

Several features of Haredi society make this regulatory dynamic especially visible. First, separation from secular society is not merely a practical arrangement but a moral and theological value. Haredi identity has historically been organized around the protection of Torah observance, rabbinic authority, communal continuity, and resistance to secularization (Almond et al., 2011; Brown, 2023; Caplan and Leon, 2024; Friedman, 1991; Almond et al., 2011; Sorotzkin, 2022). Integration is therefore often evaluated not simply as modernization, opportunity, or civic participation, but as a potential threat to spirituality and collective identity (ḥilun). This highlights the paradox of contemporary Haredi society: simultaneous dependence on state welfare systems and rhetorical rejection of civic obligations (Cahaner and Malchi, 2022).

Second, Haredi society is sustained by dense communal networks and hierarchical authority structures that shape how information, obligation, and legitimacy are evaluated (Caplan and Leon, 2024; Friedman, 1991). Rabbinic and communal authority does not merely guide private religious behavior; it also structures public meaning, including which forms of education, work, technology, media exposure, and civic participation can be morally legitimized. Recent work on Haredi relationships with professional expertise, digital media, and public institutions similarly shows how external knowledge is often filtered through internal communal authority and religious legitimacy (Hochman et al., 2024; Neriya-Ben Shahar et al., 2024; Tsuria and Campbell, 2021).

Third, the community’s relationship with the Israeli state and broader secular society embodies a durable structural ambivalence. On the one hand, Haredi identity is built around religious separation, rabbinic authority, and a moral hierarchy that places Torah study and communal continuity at the center of collective life. On the other hand, Haredi individuals and institutions increasingly rely on state systems, labor markets, higher education pathways, digital infrastructures, welfare arrangements, and public resources (Cahaner and Malchi, 2022; Malach and Cahaner, 2023; Leon, 2024). This creates a central tension between symbolic autonomy and practical dependence. Within the Homobiasos framework, such tension is precisely the kind of condition under which collective self-justification is expected to become salient: dependence can be reframed as necessity, separation as protection, and nonparticipation as a higher or alternative contribution (Ami, 2022; Leon, 2024).

The tension is especially visible across three domains examined in the present study: employment and vocational integration, education and secular curricula, and military or civic service. Employment and professional training can often be framed as responses to economic necessity and, therefore, as compatible with continued religious commitment (Blumen, 2002; Cahaner, 2023; Cahaner and Malchi, 2022; Perelman et al., 2024). Higher education, especially for women or in culturally adapted settings, may also be legitimized when presented as serving family livelihood, enabling continued Torah study, or supporting communal survival (Feldman, 2019; Freund et al., 2019; Kalagy and Braun-Lewensohn, 2019). By contrast, secular core curricula and military service are more likely to be perceived as threats to the symbolic foundations of Haredi life because they implicate authority, masculinity, national belonging, exposure to secular norms, and the boundary between Torah-centered life and Israeli civic identity (Stadler and Ben-Ari, 2003; Finkelman, 2011; Malovicki-Yaffe et al., 2023; Rosman, 2023; Stadler, 2009; Malchi, 2021).

The timing of the present study sharpened these tensions. Data were collected in July–August 2025, during a period of acute national tension. Israel was engaged in an ongoing military conflict, and public debate over Haredi conscription had intensified. National polls indicated that 84% of the Israeli public supported ending the long-standing military exemption for Haredi men, and 73% of Jewish respondents identified secular–Haredi tensions as one of Israel’s most salient internal conflicts (Times of Israel, 2024; Hiddush, 2024). Thus, this context provides a socially consequential moment in which collective justification processes are expected to be especially salient.

Accordingly, the Haredi case is not used here to portray the community as uniquely inconsistent or resistant to change. Rather, it provides a theoretically rich setting for examining bounded adaptation: pragmatic adjustment in domains that can be morally reframed as continuity, alongside resistance in domains experienced as demands for ideological transformation, secularization, or subordination to external authority.

### 1.4 The present study

The present study uses a large-scale survey of Haredi adults in Israel to examine whether patterns of attitude, subgroup variation, and open-ended narratives are consistent with the idea of collective coherence regulation. The survey assessed attitudes toward education, employment, military and civic service, communal belonging, national identity, and political representation. It also included an open-ended item that allowed respondents to elaborate, in their own words, on concerns and meanings not captured by the closed-ended questions.

The study makes three contributions. First, it provides rare empirical access to a socially bounded and relatively hard-to-sample population. Second, it uses variation across domains and subgroups to examine the structure of selective openness and resistance within Haredi society. Third, it links these patterns to a broader theoretical account of collective self-justification, suggesting how groups may preserve moral continuity while selectively adapting to social, economic, and civic pressures.

Because the study is cross-sectional and was not preregistered, it cannot demonstrate homeostatic regulation as a dynamic process over time. The empirical aim is to examine whether the observed structure of attitudes and narratives is consistent with the Homobiasos framework and whether the Haredi case can serve as a theoretically informative setting for future causal, longitudinal, and experimental research.

The central expectation is not that Haredi society uniformly rejects or uniformly embraces integration, but rather that it will exhibit a patterned form of bounded adaptation. Support should be higher where integration can be perceived as pragmatic, economically necessary, or compatible with communal continuity. Resistance should be higher where integration is experienced as a demand for ideological transformation, secularization, or subordination to external authority. Accordingly, the study was guided by three theoretically derived predictions:

*H1*: Domain-threat gradient. Openness to integration will be greater in domains that can be framed as pragmatic or instrumental, such as employment or vocational training, and weaker in domains that directly threaten symbolic boundaries, such as secular core curricula or military service. Evidence consistent with this expectation would include selective support for work or culturally bounded education alongside stronger opposition to military service or secularization. Evidence against it would include uniform support or opposition across domains regardless of their symbolic meaning.

*H2*: Symbolic-boundary expectation. Resistance will be especially strong when integration is associated with loss of communal authority, exposure to secular norms, or moral contamination. Evidence consistent with this expectation would include stronger support for culturally protected arrangements, such as gender separation, closed Haredi frameworks, rabbinic oversight, or adaptation of external institutions to Haredi values. Evidence against it would include resistance explained mainly by logistical inconvenience or material self-interest.

*H3*: Bounded-heterogeneity expectation. Differences among Haredi subgroups will reflect distinct routes to preserving coherence rather than a simple conservative–modern continuum. Some groups may show pragmatic openness in one domain while maintaining strong resistance in another. Evidence consistent with this expectation would include subgroup differences in employment, education, authority, or civic attitudes that map onto different justificatory logics. Evidence against it would include a single linear pattern in which every domain simply varies by degree of modernity.

Together, these predictions allow an evaluation of the empirical plausibility of collective coherence regulation. The Haredi case is used not to exceptionalize one community, but to illuminate a broader mechanism through which groups under pressure negotiate the boundary between stability and change.

## 2 Method

### 2.1 Participants

Data were collected in July–August 2025 through Askaria (askaria.co.il), a professional online research panel specializing in Israel’s Haredi population. Eligibility required self-identification as Haredi and being an adult resident of Israel. Participation was voluntary, anonymous, and compensated by the panel provider in accordance with its standard procedures.

The full sample included 429 respondents. The sample consisted of 247 men (57.6%) and 182 women (42.4%). The mean age was 34.0 years (SD = 11.0, median = 32; valid age responses: *n* = 414). Most respondents were married (87.9%). Stream affiliation was measured by self-identification. For descriptive purposes, respondents identified as Lithuanian/Litvish (45.5% of valid responses), Hasidic (22.9%), Sephardic/Mizrahi Haredi (20.5%), Modern-Haredi or other/hybrid Haredi affiliations, including Chabad, Breslov, Edah Haredi, general Haredi, and nonclassified responses (8.0%). For inferential analyses, because several subgroups were small, stream affiliation was recoded into four analytic categories: Lithuanian, Hasidic, Sephardic, and Modern-Others.

No post-stratification weights were applied. Because the survey included item-level nonresponse, analytic Ns vary slightly across analyses. All analyses used the maximum available valid cases for each test, and the relevant Ns or degrees of freedom are reported in the Results tables. Recruitment procedures followed the standards for hard-to-reach ideological populations (Cohen et al., 2021; Staetsky, 2022), prioritizing balance across gender, age, and region. A predetermined target of ≥400 participants was established to ensure adequate statistical power (>0.80) for medium effect sizes while accounting for realistic constraints of access and cost.

### 2.2 Design and procedure

The study used a cross-sectional online survey design. The questionnaire was administered in Hebrew through a secure Qualtrics link distributed by Askaria. The survey was developed specifically for the present project to assess attitudes, perceptions, and justificatory narratives among Haredi respondents regarding education, employment, military and civic service, collective identity, and political representation.

The full questionnaire included 56 items: 15 demographic items, 9 items on education and employment, 10 items on military/civic service, 11 items on identity and community, 10 items on political attitudes, and one open-ended item. The present article focuses on domains that were theoretically central for examining collective moral coherence under conditions of ideological tension: education, employment, military/civic service, communal belonging, and national identity. The full questionnaire, is provided in the Supplementary materials.

All items were written in Hebrew and designed to use culturally familiar language. Before data collection, item wording was reviewed through informal consultation with community informants familiar with Haredi terminology, institutions, and sensitivities. This process was intended to improve linguistic clarity and cultural appropriateness rather than to validate a standardized scale.

Most focal variables were direct attitudinal indicators. This choice reflects the applied and sociological nature of the study: the central constructs concerned explicit attitudes toward concrete social issues rather than latent clinical or personality traits. Where appropriate, multi-item indices were created; otherwise, single-item measures were analyzed as direct indicators of domain-specific attitudes.

*Education and employment.* Attitudes toward employment were assessed using five statements rated on a 1–5 agreement scale. Items addressed economic independence, the importance of acquiring a profession, encouragement of Haredi participation in the general labor market, combining Torah study with work, and the perceived social status of a Haredi person who works outside full-time Torah study. One negatively worded item was reverse-coded before computing the mean employment-attitude index. Internal consistency was acceptable (Cronbach’s *α* = 0.73). Higher scores indicate more favorable attitudes toward employment and vocational integration.

Additional education-related items assessed attitudes toward academic studies for sons and daughters, support for including core/secular subjects in Haredi educational institutions, and motivations for pursuing education or employment. The item on academic studies asked whether respondents would send sons, daughters, both, or neither to academic studies. The core-curriculum item was rated on a 1–5 agreement scale, with higher values indicating stronger support for including core studies in Haredi schools. Motivation items allowed respondents to select up to two reasons from a list, including financial need, personal interest, career development, problems with existing educational frameworks, desire to integrate into broader Israeli society, and “other.”

*Military and civic service.* Attitudes toward military service were assessed using direct items on support for a military track adapted for Haredim, willingness to recommend military or civilian service to a close family member, perceived civic obligation to contribute to the state, perceived importance of Torah study relative to military conscription, and views about who should decide whether a yeshiva student serves in the army. Several items used 1–5 agreement or support scales, whereas others were categorical or multiple-choice items. Conditions for Haredi military participation were measured through a multiple-selection item that included options such as full kashrut, closed Haredi units, Haredi commanders, combining service with Torah study, a designated Haredi corps, and adapting IDF values to Haredi values.

*Collective identity and belonging.* Identity-related measures included self-placement on a conservative–modern continuum, perceived change in Haredi society, importance of belonging to the Haredi community, sense of belonging to the State of Israel and its problems, and national pride. Items were rated on ordered response scales, typically 1–5, with higher values indicating stronger endorsement of the relevant construct. Additional items assessed consultation patterns in major life decisions, including consultation with rabbis, admorim, family members, community figures, professionals, and media sources.

*Demographics and background* var*iables*. Demographic variables included gender, age, marital status, number of children, city of residence, stream affiliation, education, employment status, income, phone type, internet use, newspaper exposure, radio exposure, and voting behavior.

### 2.3 Analytic approach

Quantitative analyses were conducted using SPSS 29. Descriptive statistics were computed for all focal variables. Group differences across Haredi streams and demographic categories were examined using chi-square tests for categorical variables and one-way ANOVAs or t-tests for continuous or ordinally treated scale variables. Where appropriate, post-hoc comparisons were used to clarify specific between-group differences. Associations between continuous variables were examined using Pearson correlations. All tests were two-tailed with *α* = 0.05.

The analyses report effect-size indicators alongside significance tests where appropriate: Cramer’s V for chi-square tests, *η*^2^ or partial *η*^2^ for ANOVAs, Cohen’s d for t-tests, and Pearson’s r for correlations. Given the exploratory and cross-sectional nature of the study and the number of group comparisons, statistical significance should be interpreted with caution. The emphasis is placed on the consistency, direction, and magnitude of patterns across theoretically related domains rather than on isolated significant findings.

At the end of the questionnaire, respondents were invited to write freely about thoughts, feelings, or general comments that had not been captured by the closed-ended questions. The prompt asked respondents: “Is there anything else you would like to share with us regarding the topics raised in the questionnaire?” This item was intentionally broad and unrestricted, allowing respondents to raise issues they considered important in their own words. The qualitative component was used as an interpretive supplement rather than as a stand-alone qualitative study. Of the 429 respondents, 134 entered some text in the open-ended field. After excluding non-substantive responses such as “no,” “thank you,” or brief comments unrelated to the research questions, 65 substantive responses were retained for thematic analysis.

The analysis followed a hybrid deductive–inductive approach. First, responses were read in full to identify recurrent themes relevant to the study’s theoretical focus on moral coherence, boundary maintenance, and justification. Second, initial codes were organized around the survey’s main empirical domains, particularly military service, education, employment, and collective identity. Third, recurring themes were refined into broader interpretive categories, including fear of secularization/hilun, reframing Torah study or volunteering as contribution, delegated responsibility to rabbinic authority, and boundary maintenance around who counts as “truly” Haredi. Finally, representative quotations were selected to illustrate themes that complemented or clarified the quantitative patterns.

All responses were originally written in Hebrew. Quotations reported in the article were translated into English by the author and checked against the original Hebrew to preserve their substantive meaning. Because the qualitative analysis served to contextualize quantitative findings rather than to establish independent prevalence estimates, no intercoder reliability statistic was calculated. To reduce overinterpretation, qualitative claims are presented as illustrative of recurrent narrative patterns and are linked explicitly to corresponding quantitative findings.

The qualitative analysis was conducted by the author. After the initial thematic coding, an academic colleague familiar with the research question reviewed the coded excerpts and representative quotations as an informal peer audit. The colleague was asked to assess whether the proposed themes were adequately supported by the raw responses and whether the selected quotations reflected the interpretive claims made in the manuscript. Points of uncertainty were discussed briefly and used to refine the interpretation. Because this procedure was intended as a credibility check rather than a formal independent coding process, no intercoder reliability statistic was calculated.

### 2.4 Ethical considerations and data availability

The study was approved by the Baruch Ivcher School of Psychology Ethics Committee at Reichman University (P_2025180, July 8, 2025). Participants provided informed consent before beginning the questionnaire and were informed that participation was voluntary and that they could stop at any point. No names or contact details were requested by the research team. The analytic dataset was de-identified before analysis and sharing. The de-identified data and study materials are available through the project’s OSF repository.

The study was not preregistered. This decision reflects the pragmatic and exploratory nature of the project rather than an omission. The primary aim was to obtain ecologically valid data from a socially enclosed and hard-to-reach population under strict access and budget constraints. Given the novelty of applying the Homobiasos framework to collective reasoning and the scarcity of comparable datasets from this population, methodological flexibility was prioritized to maximize representativeness, scope, and cultural sensitivity. All materials and de-identified data (in Hebrew) are available at https://osf.io/3n6s8/overview to ensure full transparency and replicability.

## 3 Results

### 3.1 The Israeli haredi society in 2025: sample validity and sociocultural context

Before turning to the main results, it is important to describe the sample and sociocultural context in which it was collected. This descriptive section, summarized in Table 1, clarifies the composition of the sample, documents key contextual features relevant to interpreting attitudes toward integration, and shows the degree of heterogeneity within the Haredi population. The domain-specific results are then presented in Tables 2^4^ summarizes how the findings relate to the three theoretical predictions introduced above.

**Table 1:** Sample profile and key contextual indicators.

Characteristic	Estimate	Valid N/note
Full sample	429 respondents	Adults self-identifying as Haredi; unweighted online panel
Gender	247 men (57.6%)	*N* = 429
	182 women (42.4%)	
Age	M = 34.0 (SD = 11.0, median = 32)	*n* = 414
Marital status	87.9% married	Valid percentage
Stream affiliation	Lithuanian 45.5%	Valid stream responses
	Hasidic 22.9%	
	Sephardic 20.5%	
	Modern-Others 8.0%	
Employment status	Non-Torah work 48.2%	Descriptive
	Torah-related work 16.4%	
	Full-time Kollel (men) 19.2%	
Digital exposure	Internet access 76.7%	Descriptive
	Kosher/non-smart phone 60.5%	

Percentages are valid percentages. Because of item-level nonresponse, analytic Ns vary across tests and are reported in the text or relevant tables.

**Table 2:** Education and employment attitudes by Haredi stream.

Outcome	Overall	Lithuanian	Hasidic	Sephardic	Modern/Others	Group comparison/effect
Employment attitudes index, M (SD)	3.50 (0.87)	3.18 (0.90)	3.95 (0.68)	3.55 (0.76)	3.83 (0.77)	F(3,415) = 22.24, *p* < 0.001, *η*^2^ = 0.14
Would send daughters only to academic studies, %	38.5	40.8	27.6	60.5	17.0	*χ*^2^(9) = 45.85, *p* < 0.001, V ≈ 0.19
Would send both sons and daughters, %	22.4	16.8	25.5	19.8	46.8	
Would send neither sons nor daughters, %	35.8	41.4	41.8	18.6	31.9	
Core-curriculum support, M (SD)	2.44 (1.34)	2.14 (1.29)	2.68 (1.32)	2.67 (1.31)	2.79 (1.41)	F(3,418) = 5.97, *p* < 0.001, *η*^2^ = 0.04

Means are on 1–5 scales, with higher values indicating greater support. Percentages are rounded valid percentages. The gender comparison for the employment index was also significant (men M = 3.62, women M = 3.33; F(1,417) = 11.87, *p* < 0.001, *η*^2^ = 0.03).

**Table 3:** Military and civic-service attitudes by Haredi stream.

Outcome (%)	Overall	Lithuanian	Hasidic	Sephardic	Modern/Others	Group comparison/effect
Opposed adapted military service	≈67	73.6	68.9	68.9	59.7	*χ*^2^(18) = 34.73, *p* = 0.010, V ≈ 0.17
Decision should be made by rabbis	81.8	83.3	85.7	79.1	68.8	*χ*^2^(6) = 17.65, *p* = 0.007, V ≈ 0.14
Decision should be made by the individual	13.3	12.5	8.2	17.4	19.1	—
Would recommend neither military nor civilian service	≈55	66.1	40.8	55.8	42.6	*χ*^2^(9) ≈ 50.16, *p* < 0.001, V ≈ 0.20
Would recommend civilian service only	28.0	22.4	45.9	27.9	17.0	—
Selected “adapt IDF values to Haredi values” as a condition	≈60	63.7	62.2	51.7	60.4	Descriptive
Selected “closed Haredi units” as a condition	≈58	60.6	58.2	51.7	54.2	Descriptive

Percentages are rounded valid percentages. Conditions were multiple-selection items; therefore, percentages do not sum to 100. For H2, the most diagnostic rows are the conditions emphasizing value adaptation and closed Haredi units.

**Table 4:** Mapping the results onto the three theoretical predictions.

Prediction	Key quantitative pattern	Qualitative pattern	Evaluation of these data
H1: domain-threat gradient	Employment and bounded higher education were more acceptable than core curricula or military service.	Work/study were often framed as livelihood or continuity rather than cultural integration.	Consistent, but cross-sectional data cannot establish dynamic regulation over time.
H2: symbolic-boundary expectation	Resistance was strongest where rabbinic authority, secular exposure, or military values were salient; preferred conditions emphasized Haredi values and closed units.	The IDF was described as secularizing, while Torah study and volunteering were framed as alternative forms of contribution.	Consistent with a symbolic-boundary account rather than a purely logistical account.
H3: bounded-heterogeneity expectation	Streams differed by domain: Hasidic and Modern/other respondents were more open to work; Sephardic respondents were more open to daughters’ education; Modern/other respondents showed more autonomy.	Open respondents still described limits tied to communal solidarity and boundary protection.	Partly consistent: heterogeneity was patterned, but subgroup size and panel selection limit precision.

The stream distribution was broadly consistent with existing descriptions of the main internal divisions within Israeli Haredi society (Cahaner and Malchi, 2022; Malach and Cahaner, 2023; Israel Democracy Institute, 2024). Respondents were distributed across four principal Haredi streams in proportions similar to national projections: 45.5% Lithuanian, 22.9% Hasidic, 20.5% Sephardic, and 8% Modern-Others. This alignment is critical because stream affiliation predicts distinct patterns of moral reasoning, civic engagement, and resistance to integration (Caplan and Leon, 2024). The Lithuanian stream emphasizes intellectual rigor in Talmudic study and defines sanctity through total immersion in the yeshiva world. This stream represents the most cognitively and socially insulated pole of Haredi society (Leon, 2023). The Hasidic stream is both theologically conservative and pragmatic, allowing economic participation when framed as a means of sustaining the community’s spiritual mission (Caplan and Leon, 2024; Leon, 2020). The Sephardic Haredi stream maintains deep religious commitment but is more exposed to Israeli civic life, particularly through military service networks and labor markets (Leon, 2016, 2023). Finally, the small but growing Modern streams combine religious devotion with technological use, higher education, and cautious civic engagement (Ami, 2022; Finkelman, 2011). The slight overrepresentation of Lithuanians may reflect known sampling challenges in accessing Hasidic communities but does not compromise the validity of between-group comparisons. Several smaller groups were too small for separate inferential analysis. For this reason, Modern-Haredi respondents were combined with other and hybrid Haredi affiliations in the inferential analyses. This coding decision preserves the main distinction between the large institutional streams while avoiding unstable estimates for very small subgroups.

The gender ratio (57.6% men, 42.4% women) and mean age (M = 34.0, SD = 11.0) also align with prior large-scale surveys (Staetsky, 2022). A relatively balanced gender distribution is important given documented differences in economic participation and openness to integration between Haredi men and women (Perelman et al., 2024).

Several descriptive indicators point to the coexistence of strong communal attachment with selective exposure to broader Israeli society. Most respondents reported strong or very strong belonging to the Haredi community (≈88%). At the same time, employment rates (≈65%) and the prevalence of full-time Torah study among men (≈19%) are consistent with 2022–2023 baselines (Malach and Cahaner, 2023), with a possible modest increase in economic integration. The majority reported secular employment (48.2%), suggesting bounded pragmatism (Caplan and Leon, 2024; Malach and Cahaner, 2023). Similarly, internet use (76.7%) exceeded 2022 levels (62%; Israel Democracy Institute, 2024). However, filtered “kosher” access remained dominant (55%), and smartphone use was limited (35%).

Consultation patterns further illustrate the centrality of internal communal authority (Caplan and Leon, 2024; Friedman, 1991). When asked whom they consult on significant life decisions, respondents prioritized rabbis (M = 3.76, SD = 1.19) significantly above the scale midpoint (*t*(418) = 12.98, *p* < 0.001). Family members were also rated above the midpoint (M = 3.48, SD = 1.53; *t*(405) = 7.94, *p* < 0.001). By contrast, professionals were not rated significantly above the midpoint (M = 2.91, SD = 1.32; *t*(403) = −1.43, *p* = 0.152), and media sources were rated very low (M = 1.43, SD = 0.80; *t*(399) = −39.43, *p* < 0.001). Thus, even when respondents reported practical engagement with work, technology, or state systems, major life decisions remained embedded primarily in family and rabbinic authority structures (Hochman et al., 2024; Neriya-Ben Shahar et al., 2024; Tsuria and Campbell, 2021).

Finally, the survey was conducted in July–August 2025, during a period of ongoing military conflict and renewed public debate over Haredi conscription. Public pressure around military service was therefore unusually salient. The results should consequently be interpreted as attitudes expressed under conditions of heightened civic and symbolic tension, rather than as context-free measures of general ideology.

Taken together, these descriptive findings provide the empirical background for the domain-specific analyses below. They show a sample characterized by strong Haredi belonging, continued reliance on internal authority, substantial practical exposure to work and technology, and meaningful heterogeneity across streams. These characteristics are important for evaluating whether the results show uniform resistance to integration or, as predicted by H1–H3, a patterned combination of openness, boundary maintenance, and subgroup variation.

### 3.2 Education and higher/vocational studies

#### 3.2.1 Selective openness to academic studies

Respondents expressed a cautiously positive orientation toward higher education, particularly for women (Table 2). When asked whether they would send their children or close relatives to academic studies, 38.5% supported women’s participation, compared with only 2.1% who would send men only. An additional 22.4% would allow all genders, while approximately 35% rejected academic studies for both. Thus, openness to academic study was clearly gendered and bounded rather than general. This pattern is consistent with previous findings (Friedman, 1991; Keren-Kratz, 2025) and H1 because support was higher where education could be framed as family livelihood or culturally bounded adaptation than where it involved broader gender asymmetry and status change.

Differences across streams were significant (*χ*^2^(9) = 45.85, *p* < 0.001). Sephardic respondents exhibited the highest support for women’s education (≈61%) and the lowest total opposition (≈17%), whereas Hasidic and Lithuanian participants were significantly less open (≈41–43% opposition). However, Hasidic were more open to sending men (26.8%), while Lithuanians preferred sending only women (39.3%). Modern-Others displayed the most supportive stance, with only 30.4% opposing participation by all genders. These results are consistent with broader patterns of adaptive stability observed across Haredi society (Cahaner and Malchi, 2022; Malach and Cahaner, 2023).

#### 3.2.2 Persistent resistance to core (secular) curricula

When asked about introducing core subjects (e.g., mathematics, English, and science) into Haredi schools, a clear majority expressed opposition. More than half of respondents (53.3%) disagreed or strongly disagreed, whereas only 23.8% supported integration. The mean score (M = 2.44, SD = 1.34) was significantly below the neutral midpoint (*t*(420) = −8.63, *p* < 0.001), indicating a broadly medium level of resistance even among more modern subgroups.

Differences across streams were statistically significant (*F*(3,418) = 5.97, *p* < 0.001). Lithuanian respondents showed the strongest opposition (M = 2.14, SD = 1.29), consistent with their traditional emphasis on Torah study as an exclusive epistemic domain. Hasidic (M = 2.68, SD = 1.32) and Sephardic (M = 2.67, SD = 1.31) respondents showed lower resistance, while Modern showed the weakest (M = 2.72, SD = 1.44).

These results show a clear distinction between forms of education that can be treated as pragmatic adaptation and those that implicate the formal socialization of children (Caplan and Leon, 2024; Malach and Cahaner, 2023; Perelman et al., 2024; Keren-Kratz, 2025). In support of H1, openness was higher for bounded adult higher or vocational education than for secular core curricula. In support of H2, the stronger resistance to core curricula indicates that education becomes more contested when it touches communal authority, cultural exposure, and the reproduction of group boundaries.

### 3.3 Employment and economic roles

#### 3.3.1 General attitudes toward employment

General attitudes towards employment, averaged across the five items, were moderately positive (M = 3.50, SD = 0.87) and significantly above the neutral midpoint (*t*(418) = 11.75, *p* < 0.001; Table 2). This indicates a general acknowledgment that economic self-reliance is desirable, though the intensity of endorsement varied markedly across subgroups (*F*(3,415) = 22.24, *p* < 0.001, *η*^2^ = 0.14). Hasidic respondents expressed the most positive attitudes (M = 3.95, SD = 0.68), followed by Modern-Others (M = 3.83, SD = 0.77) and Sephardic respondents (M = 3.55, SD = 0.76). Lithuanian respondents showed the lowest mean score (M = 3.18, SD = 0.90).

A clear gender effect also emerged (*F*(1,417) = 11.87, *p* < 0.001, *η*^2^ = 0.03). Men expressed significantly stronger approval of work (M = 3.62, SD = 0.83) than women (M = 3.33, SD = 0.91). These gender and stream differences are consistent with previous research (Malach and Cahaner, 2023; Perelman et al., 2024) and H3: openness to work did not vary along a single conservative–modern continuum, but differed by subgroup location and by the social meaning of work within each subgroup.

Importantly, the Hasidic sector’s relatively liberal attitude toward employment is consistent with previous sociological findings (Leon, 2020; Cahaner and Malchi, 2022; Caplan and Leon, 2024). Despite its theological conservatism, Hasidic society tends to adopt a pragmatic stance toward work and economic participation. The finding that the highest support for employment was among Hasidic respondents aligns with this functional traditionalism, in which livelihood is morally framed as sustaining Torah life rather than contradicting it.

#### 3.3.2 Motivations for work and study

When asked about their two most important motives for pursuing education or employment, nearly 80% of respondents cited economic pressure or financial necessity as their primary reason. In contrast, only about 30% mentioned personal interest or career development, and 16.8% mentioned problems with traditional frameworks. Merely 9.8% selected integration into broader Israeli society as a motivating factor, while 4.4% chose “other.” These patterns were consistent across all streams.

These findings indicate that work and nonreligious higher studies are usually framed as pragmatic necessities rather than as intrinsic moral or national values (Blumen, 2002; Cahaner, 2023; Freund et al., 2019). Even when respondents endorsed combining Torah study with livelihood, this was typically envisioned as part-time or religiously embedded work, not as full cultural integration. This pattern is consistent with H1 and H2: integration was more acceptable when attached to economic need and less acceptable when it was framed as cultural assimilation.

### 3.4 Conscription and civic duty

Military service showed the strongest pattern of resistance among the domains examined (Table 3). A central declared barrier to Haredi enlistment is the fear of religious erosion through exposure to secular society (Malovicki-Yaffe et al., 2023; Stadler and Ben-Ari, 2003). In the present survey, when respondents selected the two most important conditions that would enable Haredi participation in military service, nearly 60% chose adaptation of the military to Haredi values and closed Haredi units. By contrast, more technical accommodations, such as maintaining a kosher kitchen, having Haredi commanders, or combining military service with Torah study, were chosen by fewer respondents. This distribution is consistent with H2 because the most frequently selected conditions concerned symbolic and communal protection rather than logistical convenience alone.

#### 3.4.1 Authority and decision-making

When asked who should decide whether a yeshiva student serves in the army, 81.8% of respondents attributed the decision to the rabbi. In comparison, only 13.3% chose the individual himself, and 5% selected the family. This distribution shows that rabbinic authority remained the dominant source of legitimacy for decisions about military service.

Variations across streams and socioeconomic strata nevertheless emerged. Although the rabbi was the dominant figure across all streams, Modern-Others (19.1%) and Sephardic (17.4%) respondents assigned greater independence to the individual than Hasidic (8.2%) and Lithuanian respondents (12.5%; *χ*^2^(6) = 17.65, *p* = 0.007, V ≈ 0.14). Respondents with higher incomes were also more likely to favor individual autonomy (*χ*^2^(10) = 35.42, *p* < 0.001). This pattern should be interpreted descriptively, but it is consistent with H3 because subgroup differences appeared in one domain of authority while broad resistance to military service remained visible across groups.

#### 3.4.2 Attitudes toward military service

Roughly 67% rejected even a Haredi-adapted service model, with only 23% expressing partial or full support. Still, this figure represents a modest change relative to the nearly 80% rejection rate reported in earlier surveys (Jewish People Policy Institute, 2024). Opposition peaked among Lithuanian (73.6%) and Sephardic (68.9%) respondents, declined among Hasidic (68.9%), and was lowest among Modern-Others (59.7%). These differences were significant (*χ*^2^(18) = 34.73, *p* = 0.01). Gender introduced another layer: men were somewhat more supportive of enlistment (45.7% opposition) than women (60.2%; *χ*^2^(6) = 14.81, *p* = 0.022). This gender gap may reflect men’s direct stake in the issue and the emerging ambivalence among younger cohorts. Yet across all groups, ideological resistance remained the normative baseline.

#### 3.4.3 A parallel belief: the duty to contribute

When asked whether they agreed with the statement that every citizen, regardless of sector, should contribute to the state through military or civilian service, the distribution shifted. More than half of respondents (52.6%) disagreed, almost 38% agreed at least partially, and less than 10% were uncertain. Thus, abstract civic contribution received more endorsement than military service itself. This gap indicates that some respondents accepted the general value of contribution while rejecting its institutional expression through the army.

#### 3.4.4 Recommendation to a family member

The gap between abstract contribution and concrete action reappeared when respondents were asked whether they would recommend close family members to enlist in military or civilian national service. Only 14% would recommend both, 28% would recommend civilian service only, and a majority (approximately 55%) would advise against both. While nearly 23% personally supported military service, fewer than 16% would recommend others to enlist. Similarly, although about 70% opposed military service, nearly 85% said they would not recommend it. Thus, willingness to endorse contribution in principle was higher than willingness to recommend participation in practice.

The qualitative responses help clarify the meaning of this gap. Many respondents justified nonparticipation by redefining contribution through Torah study, religious continuity, or communal volunteering. Statements such as “We all contribute—each in his own way,” “Torah study is also protection for the nation,” and “We contribute more than any other sector” illustrate a narrative pattern in which refusal of military participation is paired with affirmation of alternative contribution. This pattern is consistent with H2 because resistance was linked to the preservation of religious authority and moral standing rather than to material self-interest alone.

### 3.5 Belonging and identity

Behavioral domains such as higher education, employment, and civic participation reveal how ideology is negotiated through action. Belonging, by contrast, captures the emotional and symbolic substrate that enables these negotiations to remain coherent. For the Haredi population, identity and belonging constitute not merely social affiliations but moralized commitments embedded in a sanctified communal order (Almond et al., 2011; Caplan and Leon, 2024; Keren-Kratz, 2025). Within this framework, belonging situates individuals vis-à-vis both the intra-communal hierarchy and the broader civic sphere.

#### 3.5.1 Perceived modernity and conservatism

When asked to position themselves on a scale from conservative to modern, 52.0% identified as conservative, 38.2% as “both conservative and modern”, and only 8.5% as modern. This distribution supports recent sociological typologies distinguishing “Haredi modernity” from broader religious modernism (Caplan and Leon, 2024; Malchi, 2021). The relatively high “both/and” category aligns with a growing hybrid identity within the community that maintains communal belonging and symbolic orthodoxy while adopting selective flexibility in practice (Finkelman, 2011; Malchi, 2021; Malach and Cahaner, 2023).

Stream differences in self-placement were statistically significant (*χ*^2^(6) = 23.23, *p* < 0.001). Each stream showed a different configuration of conservatism, hybridity, and selective openness. Lithuanian respondents remained highly conservative. Hasidic respondents, while theologically conservative, showed evidence of practical engagement with a high proportion of “both/and” (Caplan and Leon, 2024; Leon, 2020). Sephardic respondents occupied an intermediate position, with a relatively balanced share choosing the conservative and “both/and” categories. The Modern-Others group showed the highest rate of explicitly modern self-placement.

When asked to evaluate the broader trajectory of Haredi society on that scale, more than half (55.1%) perceived Haredi society as becoming more modern, whereas less than a quarter (24.3%) viewed it as becoming more conservative. The remaining respondents believe nothing has changed in Haredi society. This dual perception of self as conservative and society as modernizing may suggest that respondents acknowledge collective change without internalizing it as a personal transformation. The individual remains morally anchored while social change is projected outward onto the collective (Ellemers, 2017). This externalization pattern echoes findings in Sections 3.2, 3.3, where participation in higher education or professional training may reflect a pragmatic necessity rather than an ideological realignment.

This pattern of results suggests that exposure to modernity may not necessarily be perceived as erosion but as managed evolution, a change under the guardianship of communal authority (Friedman, 1991; Caplan and Leon, 2024). The paradox of being personally conservative while recognizing collective modernization may help keep moral continuity through adaptive reinterpretation and selective exclusion.

#### 3.5.2 National belonging

Respondents rated their sense of belonging to the State of Israel and its challenges. The mean score (M = 3.42, SD = 1.27) was significantly higher than the scale midpoint (*t*(417) = 6.81, *p* < 0.001), indicating that Haredi participants experience a moderate yet statistically significant sense of attachment to the Israeli state. Still, the mean sense of belonging to the Haredi community (after coding the reported results in Section 3.1.2 to a 1–5 scale) was markedly higher (M = 4.56, SD = 0.84). A paired-samples t-test confirmed that communal belonging was significantly stronger than state belonging (*t*(416) = 17.94, *p* < 0.001). Thus, while many respondents express an affective connection to the national entity, this connection remains secondary to communal identification (Caplan and Leon, 2024).

Correlational analyses revealed a moderate positive association between belonging to the state and the questions reported in Sections 3.4.2 and 3.4.3: support for civic contribution (*r* = 0.42, *p* < 0.001) and support for military service (*r* = 0.33, *p* < 0.001). These findings may suggest that emotional attachment aligns only with selective domains of civic engagement (Turner et al., 1979; Roccas and Brewer, 2002). This may allow individuals to sustain parallel commitments to distinct, at times conflicting, collective frameworks. Within this configuration, national belonging can coexist with primary allegiance to the religious collective.

#### 3.5.3 National pride

Respondents also rated their agreement with the statement “I am proud to be Israeli.” The mean score (M = 3.26, SD = 1.36) was significantly higher than the scale midpoint (*t*(419) = 3.996, *p* < 0.001), indicating a modest yet statistically significant sense of national pride among the Haredi population. Sephardi respondents reported the highest mean (M = 3.92, SD = 1.12), followed by Modern-Others (M = 3.36, SD = 1.47). The Hasidic had a slightly lower mean (M = 3.32, SD = 1.28), whereas the Lithuanian reported the lowest mean (M = 2.92, SD = 1.35; *F*(3, 416) = 11.977, *p* < 0.001).

The correlation between pride and state belonging was strong (*r* = 0.65, *p* < 0.001), reflecting substantial conceptual overlap between the two constructs. Pride also correlated moderately with support for civic service (*r* = 0.41, *p* < 0.001) and military service (*r* = 0.33, *p* < 0.001), paralleling the behavioral associations reported in the previous section. Yet, belonging and pride represent distinct facets of national attachment. Belonging entails perceived inclusion and shared fate, a cognitive-affective identification with the collective and its challenges. Pride, by contrast, constitutes a symbolic and evaluative affirmation of that collective, often shaped by normative expectations and social desirability (Schatz et al., 1999).

The findings suggest that both belonging and pride are anchored more strongly in symbolic and normatively constrained values than in pragmatic or institutional engagement. National attachment may be filtered through communal boundaries and ideological scripts, resulting in bounded participation that endorses the principle of civic duty while resisting its required behavioral expression (Brown, 2023; Friedman, 1991). Accordingly, Haredi respondents exhibit strong inward moral belonging (to the community) alongside moderate outward civic belonging (to the state).

### 3.6 Qualitative narratives: moral framing and cognitive dissonance repair

Thematic analysis identified three dominant patterns reflecting collective strategies of moral coherence and justification under ideological tension.

#### 3.6.1 A fear of hilun

A dominant moral narrative among respondents portrayed military enlistment as both an existential and spiritual threat to the Haredi collective. Participants frequently framed the Israel Defense Forces (IDF) not as a civic institution but as an agent of secularization and moral contamination. Typical statements included: “The army cancels Halacha,” “Those who receive orders from others are slaves, which is against Halacha,” and “The army of the secular state,” or “a fusion chamber.” Such expressions delineate a categorical moral boundary rather than a pragmatic concern, recasting the IDF as antithetical to divine authority and communal sanctity. This framing is consistent with research showing that Haredim consistently construe the army as a locus of spiritual risk and moral pollution rather than civic participation (Caplan and Leon, 2024; Malchi, 2021; Almond et al., 2011). Further studies document the stigma and social exclusion faced by enlistees, reinforcing the perception that integration entails spiritual compromise (Malovicki-Yaffe et al., 2023; Stadler et al., 2008).

This was not limited to the conservative sectors. As one Modern-Haredi respondent explained, “Even the modern ones will not enlist, because if they do, the traditionalists will be left alone in the fight against secularization.” This insight, which mirrors the quantitative findings in Section 3.4.2, suggests that sustaining group coherence may require self-limiting adaptation even among those most open to civic engagement (Cooper, 2024).

#### 3.6.2 Collective pride and the reframing of contribution

Many respondents expressed a countervailing sense of collective pride, a conviction that the Haredi community already contributes to Israeli society more than any other sector. This moral self-affirmation may help transform external accusations of nonparticipation into evidence of moral superiority (Halevy et al., 2025; Hochman et al., 2021).

Participants frequently stated that “the Haredi public contributes the most to the country—economically, spiritually, and through volunteer work.” Others argued, “We are being prosecuted for studying Torah.” Respondents also cited widespread communal volunteering in organizations such as Magen David Adom, United Hatzalah, ZAKA, and Ezer Mizion, portraying these acts as both spiritually superior and socially indispensable. This discourse may reflect collective moral licensing, a process through which groups maintain moral self-regard by emphasizing virtue in alternative domains (Monin and Miller, 2001; Ellemers, 2017).

Private correspondence reinforced this dynamic. One respondent recounted that a senior rosh yeshiva discouraged volunteering during wartime: “Why help them? They’ll curse you and still call you draft dodgers.” Another insisted: “Our contribution to the nation is already a thousand times greater than yours.” This combination of defensive pride and motivated reasoning may protect collective self-worth by redefining virtue relationally, against the secular out-group, rather than through behavioral conformity. This narrative, in which “Torah study protects the nation more than the army,” may reframe nonparticipation as a symbolic contribution that reconciles communal sanctity and civic belonging.

#### 3.6.3 Moral conflicts and delegated responsibility

Respondents frequently articulated positions that both reject and reproduce theological contradictions. As described in Section 3.6.1, many portrayed the army as a forbidden space of spiritual enslavement. Yet this framing stands in contrast to core religious imperatives of communal duty and to the findings in Sections 3.1.2 and 3.4.1, which underscore the community’s extensive deference to rabbinic authority. This paradox was captured in statements such as: “No rabbi allows enlistment under any circumstances,” “You value personal opinions, but a true Haredi follows his rabbis, not his own mind,” and “Everyone who has a rabbi consults about everything and believes everything is from God.”

Contradictions also surfaced in more pragmatic moral claims, such as, “Enlistment cannot be forced,” “A Jew cannot be a slave to anyone,” “Haredim who connect with seculars are simply not Haredim,” and the ambivalent declaration, “I will never enlist. I am a proud Haredi—please find me a job in high-tech.” Together, these expressions portray a framework that legitimizes asymmetry in ethical expectations: coercion is condemned when applied internally (“recruitment cannot come through force”) yet accepted when externally justified (e.g., the national prohibition on public transportation during Shabbat). Similarly, integration is morally endorsed when framed as an economic necessity but rejected when framed as a national obligation.

These qualitative patterns illustrate how respondents managed competing imperatives of obedience and autonomy, separation and adaptation. Rather than resolving these tensions through a single consistent position, participants often used religious authority, collective contribution, and boundary protection to make the tensions intelligible. This qualitative pattern complements the quantitative results and provides additional evidence consistent with H1–H3.

## 4 Discussion

The present study examined whether attitudes among Israeli Haredi respondents toward education, employment, and military or civic service are consistent with the idea of collective coherence regulation. Across domains, the findings revealed a patterned form of bounded adaptation: pragmatic openness where integration could be framed as necessity, continuity, or service to the community, alongside strong resistance where integration was experienced as threatening religious authority, symbolic boundaries, or the moral foundations of Haredi identity. Because the study is cross-sectional and exploratory, these findings cannot demonstrate a dynamic homeostatic mechanism over time. They do, however, provide converging quantitative and qualitative evidence consistent with the Homobiasos framework and with the broader claim that groups may preserve moral coherence by reinterpreting contradiction rather than eliminating it.

Respondents were not uniformly opposed to integration and showed bounded adaptation across domains. Employment and higher or vocational education received substantially more acceptance than secular core curricula or military service. This suggests that resistance is not simply a general rejection of modernity, state institutions, or practical adaptation. Rather, openness depended on whether the domain could be interpreted as compatible with communal continuity. Consistent with the first prediction, openness was greater in domains that could be framed as pragmatic or instrumental, and weaker where integration was associated with symbolic transformation.

This was especially clear in attitudes toward education. Academic participation was far more acceptable for daughters than for sons, and openness to women’s education appeared morally intelligible when framed as supporting family and Torah study, rather than as individual self-realization or secular integration. This pattern echoes prior work on gendered adaptation in Haredi society and suggests that educational change can be legitimized when it serves existing communal roles rather than challenges them (Friedman, 1991; Feldman, 2019; Freund et al., 2019; Kalagy and Braun-Lewensohn, 2019; Keren-Kratz, 2025; Perelman et al., 2024). In this sense, higher education does not necessarily signal ideological transformation. It may function as a bounded form of modernization: behavioral adaptation under a stable moral narrative.

By contrast, secular core curricula were much more strongly resisted. Core curricula are not merely another form of education. They touch the symbolic infrastructure of the community: who has authority over children, what knowledge counts as legitimate, and whether secular epistemic frameworks are allowed to enter the protected space of Haredi socialization. This pattern is consistent with previous accounts of Haredi boundary maintenance and with the idea that education becomes especially contested when it implicates communal authority, exposure to secular knowledge, and the reproduction of group identity (Brown, 2023; Caplan and Leon, 2024; Finkelman, 2011; Neriya-Ben Shahar, 2017).

A similar pattern appeared in employment. Respondents expressed moderately positive attitudes toward work and economic self-reliance, but the dominant motivational frame was financial necessity rather than cultural integration. Thus, the willingness to work or acquire a profession does not necessarily reflect a desire to assimilate into wider Israeli society. Instead, it can be interpreted as a practical adjustment that allows individuals and families to survive economically while preserving the broader moral architecture of Haredi life. This is precisely the kind of limited modernization predicted by the Homobiasos framework. Adaptation is tolerated when it can be narrated as continuity, necessity, or service to Torah-centered life (Blumen, 2002; Cahaner, 2023; Cahaner and Malchi, 2022; Malach and Cahaner, 2023; Perelman et al., 2024).

Military service showed the strongest form of resistance, even when respondents were asked about an adapted Haredi track. The conditions for possible participation emphasized the protection of values and separation over technical accommodation. Respondents prioritized adapting military frameworks to Haredi values and maintaining closed Haredi units. These preferences suggest that the central concern is not merely logistics, kashrut, or convenience, but the fear that military participation could expose individuals to secular norms, weaken rabbinic authority, and redefine the meaning of Haredi masculinity and citizenship (Stadler and Ben-Ari, 2003; Malovicki-Yaffe et al., 2023; Rosman, 2023; Stadler, 2009; Malchi, 2021).

The role of rabbinic authority further supports this interpretation. Most respondents believed that rabbis, rather than individuals or families, should decide whether a yeshiva student serves in the army. This pattern reflects more than obedience as a behavioral norm. It shows how moral agency itself is embedded in a collective authority structure (Caplan and Leon, 2024; Friedman, 1991). In a community where legitimate action depends on religious authorization, individual choice may be experienced not as autonomy but as exposure to moral danger. Therefore, integration is acceptable only when it is mediated by internal institutions capable of preserving symbolic security (Caplan and Leon, 2024; Friedman, 1991; Neriya-Ben Shahar et al., 2024; Tsuria and Campbell, 2021).

At the same time, the findings do not show a simple absence of civic morality. Many respondents endorsed the abstract idea that citizens should contribute to the state, yet opposition to military service and reluctance to recommend it to a close family member remained high. This gap between abstract contribution and concrete institutional participation suggests that civic obligation should be reinterpreted before it can become compatible with Haredi moral order (Ami, 2022; Leon, 2024). The qualitative responses repeatedly reframed Torah study, religious continuity, and communal volunteering as alternative or even superior forms of contribution. Statements such as “we all contribute, each in his own way,” “Torah study is also protection for the nation,” and “we contribute more than any other sector” reveal a logic of contribution without institutional conformity. The civic demand to contribute is not rejected outright; it is redefined in metaphysical, communal, and religious terms.

This pattern is consistent with the second prediction. Resistance was strongest where integration was linked to secular exposure, loss of authority, or moral contamination (Caplan and Leon, 2024; Malach and Cahaner, 2023). The qualitative data further suggest that military service was often framed as a threat to religious continuity, whereas nonparticipation was reframed as faithfulness, protection, or a different form of civic service. Within the Homobiasos framework, this can be understood as collective self-justification under identity threat. The group preserves its moral standing not by denying the value of contribution, but by redefining what contribution means. This interpretation is consistent with broader research on self-serving justification, moral licensing, and motivated moral reasoning, in which individuals and groups preserve moral self-regard by emphasizing alternative domains of virtue when their conduct is challenged (Ayal et al., 2015; Barkan et al., 2015; Ellemers, 2017; Halevy et al., 2025; Hochman et al., 2021; Monin and Miller, 2001; Shalvi et al., 2015).

The data also support recent sociological typologies distinguishing “Haredi modernity” from broader religious modernism (Caplan and Leon, 2024; Malchi, 2021), pointing to a hybrid identity of Haredim who maintain communal belonging and symbolic orthodoxy while adopting selective flexibility in practice (Malchi, 2021; Malach and Cahaner, 2023; Finkelman, 2011). Hasidic respondents were relatively open toward employment, consistent with a tradition of pragmatic economic engagement (Caplan and Leon, 2024; Leon, 2020), yet they did not necessarily show parallel openness across all domains. Lithuanian respondents were more resistant in some educational and military domains, consistent with a stronger emphasis on Torah study and rabbinic authority (Leon, 2023). Sephardic respondents often occupied an intermediate position (Leon, 2016, 2023), and Modern-Others were generally more open but still retained clear boundaries around military service and communal legitimacy.

This patterned heterogeneity highlights that the Haredi society is not homogeneous in views or motives. The findings suggest that each stream may preserve coherence through different routes. For some, coherence is maintained through stronger boundary protection; for others, through pragmatic accommodation; and for others, through hybrid identities that combine conservative belonging with selective openness. This is consistent with the third prediction. Subgroup differences did not simply map onto a single conservative–modern scale, but reflected different ways of preserving continuity under social change.

The difference between personal and collective conservatism reinforces this point. Many respondents described themselves as conservative while also perceiving Haredi society as becoming more modern. This apparent contradiction suggests that respondents can acknowledge collective change without experiencing themselves as personally transformed. Change is projected onto the social environment, while the moral self remains anchored. Such a pattern is consistent with coherence regulation: modernization can be recognized, but it is managed in ways that protect the continuity of identity (Ellemers, 2017; Jost and Banaji, 1994; Leach et al., 2007; Tavris and Aronson, 2007).

A related mechanism may be boundary redefinition. Some respondents did not interpret adaptation as change within Haredi society, but as evidence that those who adapt are no longer fully Haredi. By treating integrative or modernizing individuals as marginal, exceptional, or not “truly” Haredi, the group can acknowledge behavioral change while preserving the perceived purity of the collective self-definition. In this sense, modernization is sometimes reclassified as exit rather than transformation. This mechanism has been documented in research on religious and ideological boundary maintenance and helps explain how collective identity can remain stable even while practices diversify (Almond et al., 2011; Brown, 2023; Hornsey and Hogg, 2000; Lamont and Molnár, 2002; Roccas and Brewer, 2002).

Taken together, the results suggest that Haredi responses to integration are best understood not as uniform resistance, hypocrisy, or straightforward modernization, but as a system of collective coherence regulation. A community organized around strong symbolic boundaries must continually negotiate between practical dependence and ideological autonomy (Cahaner and Malchi, 2022; Hochman et al., 2025). Employment, education, technology, welfare, and state institutions are increasingly necessary for everyday life, yet full normative integration into secular Israeli society remains morally threatening (Almond et al., 2011). The observed solution is not total rejection or full transformation, but reinterpretation.

This provides initial support to the extension of Homobiasos from individual moral cognition to collective life by showing how groups may preserve a shared sense of virtue under conditions of social strain. At the individual level, biased reasoning and motivated reinterpretations can help people sustain a livable moral self when values and actions conflict. At the collective level, shared narratives can perform a similar regulatory function (Leon, 2024). Dependence can be reframed as necessity; separation as protection; work as support for Torah; women’s education as family responsibility; and nonparticipation in military service as spiritual or communal contribution. Still, the data do not allow testing of a dynamic homeostatic process, nor do they rule out alternative explanations. Social desirability, online-panel selection, socioeconomic differences across streams, and the unusual political and military context of July–August 2025 may all have influenced responses. However, the convergence across closed-ended attitudes, subgroup differences, and open-ended narratives strengthens the claim that the findings are consistent with a coherence-preserving mechanism rather than with a purely material, logistical, or informational explanation.

More broadly, the findings suggest that conflicts between groups facing identity-threatening change are often not reducible to a confrontation between one morally innocent and one morally deficient side. In many polarized conflicts, opposing groups may each defend morally meaningful concerns while also engaging in motivated distortions that protect their own coherence. Research on social identity, attribution bias, motivated reasoning, and identity-protective cognition shows that groups tend to experience their own position as principled, contextualized, and morally necessary, while interpreting the other side’s position as irrational, selfish, hypocritical, or morally corrupt (Ditto et al., 2019; Hewstone, 1990; Jost and Banaji, 1994; Kahan, 2013, 2016; Kunda, 1990; Pettigrew, 1979; Pronin et al., 2002; Tajfel and Turner, 1986). From the perspective of Homobiasos, this does not imply that all claims are equally valid, nor that harmful practices should be excused. Rather, it suggests that many resistant or morally problematic positions are sustained because they help individuals and groups remain intelligible to themselves as decent, loyal, and coherent moral actors. This distinction is important for understanding why simply showing each side that it is wrong is unlikely to resolve such conflicts. When correction is experienced as an attack on identity, dignity, or moral standing, it can increase rather than reduce defensive reasoning (Kahan, 2013, 2016; Nyhan, 2021; Taber and Lodge, 2006; Walter et al., 2019; Wittenberg and Berinsky, 2020). In such cases, the psychological obstacle is not only an informational error but also a threat to coherence. Groups may reject accurate information, resist fair demands, or reinterpret obligations not because they lack moral concerns, but because accepting the demand in its current form would make their existing moral identity feel compromised.

Crucially, while the empirical focus of this study was the Israeli Haredi community, the theoretical claim is not limited to it. The Haredi case is used here as a socially consequential setting in which a more general process becomes visible: groups facing identity-threatening demands may respond not simply by accepting or rejecting change, but by trying to preserve moral coherence while negotiating the boundaries of adaptation. Thus, the broader contribution of the study lies in showing how collective self-justification may operate in any community whose identity, authority structures, or moral self-understanding are challenged by social, political, or institutional change.

### 4.1 Applied implications: from defensive regulation to moral repair

The applied implication of these findings is that integration policies must distinguish between correction and repair. Correction asks which belief is false and how it can be changed. Repair asks what moral identity is threatened by change and how contested obligations can be reconstructed so that they remain recognizable as legitimate within the group’s moral world. If resistance is treated only as ignorance, civic selfishness, or lack of rationality, external pressure may intensify the very defensive regulation it seeks to overcome. If resistance is understood as a coherence-preserving response, however, policy can be designed to enable adaptation without making it feel like symbolic surrender (Danbold and Huo, 2022; Hornsey and Hogg, 2000; Shnabel and Nadler, 2008). One potential solution is moral repair. The data show that Haredi respondents were not closed to all forms of change. They were more open when work, education, or contribution could be framed as continuity, necessity, or service to the community, and more resistant when change implied secularization, loss of rabbinic authority, or subordination to external moral judgment. Moral repair, in this sense, is the applied counterpart of collective coherence regulation. Defensive regulation narrows the moral field by allowing each group to preserve its own virtue while dismissing the other side’s claims. Integrative regulation expands the moral field by enabling each group to recognize that the other side may also be protecting real moral concerns, even when its conclusions remain contested (Gross et al., 2013; Halperin, 2014; Halperin et al., 2011, 2017; Noor et al., 2012; Shnabel and Nadler, 2008).

For Haredi–secular relations in Israel, this means that the conflict over education, work, and military service should not be framed only as a battle between contribution and evasion, or between coercion and religious survival. Each framing captures something real while concealing something important. Haredi resistance may reflect genuine fears of secularization, loss of communal authority, and damage to Torah-centered identity. At the same time, secular demands for contribution reflect genuine concerns about fairness, shared risk, public resources, and the moral burden carried by serving families. Both sides protect morally meaningful values, and both can distort those values when they become totalizing. Haredi claims of spiritual contribution can become a way to evade concrete civic obligations; secular claims of equality can become a way to humiliate or delegitimize Haredi identity. The practical challenge is to create arrangements that are both genuinely contributory and morally inhabitable.

Accordingly, adapted service, civic-service, educational, or employment frameworks should not be understood merely as technical accommodations. Their success depends on whether they can satisfy two moral conditions at once. Haredi participants must preserve dignity, religious continuity, and a sense that contribution does not equal assimilation. Secular Israelis must experience integration as a real contribution rather than a symbolic participation. Moral repair requires mutual reframing rather than unilateral accommodation.

A famous story of Rabbi Aryeh Levin and the disputed coat illustrates this logic. In the story, a father and son each claim a single coat for themselves. Rabbi Levin asks them to return only after each can explain why the other needs it. Once each recognizes the other’s need, he provides another coat and explains that when each claimed ownership, he, too, claimed ownership; when each was willing to give, he, too, was willing to give. The story captures the psychological movement from defensive regulation to moral repair. The solution does not emerge because one side proves the other side wrong. It emerges when each side can preserve its own moral identity while making room for the moral reality of the other. This logic follows directly from the current results. Respondents resisted change when it threatened symbolic boundaries, but showed more openness when change could be integrated into existing moral narratives. The applied lesson is therefore not that moral repair replaces policy, law, or civic obligation. Rather, it specifies a psychological condition under which such obligations are more likely to become socially livable. Change is most likely to endure when it does not require groups to abandon coherence but enables them to reconstruct it in a broader, more cooperative form.

### 4.2 Limitations and future directions

Several limitations should be acknowledged. First, the study is cross-sectional and was not preregistered. It can identify patterns consistent with collective coherence regulation, but it cannot demonstrate dynamic homeostatic adjustment over time. Similarly, the applied implications developed in Section 4.1 are theoretically and empirically grounded in the present findings, but they were not themselves tested as interventions. Claims about identity-safe framing, culturally protected integration, or mutual reframing should therefore be treated as hypotheses for future applied research rather than as established policy solutions. Before broad implementation, such approaches should be evaluated through experimental, longitudinal, or field-based designs that examine whether they actually increase openness, reduce defensive resistance, and preserve perceived communal dignity.

Second, the sample was recruited through an online Haredi panel. Although this enabled rare access to a hard-to-sample population and produced broad coverage across streams, it may overrepresent respondents who are more digitally connected, more willing to answer surveys, or more open to engagement with external institutions. This concern is especially relevant given the relatively high level of internet use in the sample. Future studies should combine online panel data with offline recruitment, institutional sampling, and targeted outreach to more insulated communities.

Third, several constructs were measured with direct single-item indicators. This choice was appropriate for concrete sociopolitical attitudes and improved cultural clarity, but it limits psychometric precision. Future work should develop and validate multi-item measures of perceived identity threat, collective coherence, rabbinic authority, moral contribution, and willingness to support bounded forms of integration.

Fourth, the qualitative analysis served as an interpretive supplement rather than a stand-alone qualitative study. The open-ended responses enriched the interpretation of the quantitative patterns, but they should not be treated as prevalence estimates. Future qualitative work should use interviews, focus groups, and formal multi-coder procedures to examine how Haredi respondents narrate work, education, military service, and civic belonging in greater depth.

Fifth, the political and military context may have intensified attitudes toward conscription and civic duty. The survey was conducted during a period of acute national conflict and heightened public debate over Haredi military service. The findings may therefore reflect attitudes under identity threat rather than stable baseline positions. This context is theoretically meaningful for Homobiasos, because identity threat is precisely the condition under which coherence-preserving justification should become salient, but it also requires caution in generalization.

Future research should test the framework prospectively and experimentally. Studies could manipulate whether employment, education, or service is framed as secular integration, economic necessity, communal continuity, or shared contribution. If the Homobiasos interpretation is correct, identity-safe framings should increase openness more than purely instrumental or coercive framings, especially among respondents who report strong communal belonging. Comparative studies should also examine whether similar mechanisms appear in other ideological or religious groups, including secular publics facing threats to autonomy, national groups facing status loss, or political communities responding to moral criticism. Such research would clarify whether collective coherence regulation is specific to the Haredi case or reflects a broader mechanism through which groups negotiate the boundary between adaptation and identity protection.

### 4.3 Conclusion

The present findings suggest that Haredi responses to integration are best understood not as a uniform rejection of modernity, but as bounded adaptation shaped by perceived moral threat. Openness was greater where work, vocational training, or women’s academic education could be framed as economic necessity, family support, or continuity with communal life, and resistance was stronger where secular core curricula or military service were experienced as threats to rabbinic authority, exposure to secular norms, gendered and communal roles, and the symbolic boundaries of Haredi identity. Together with the qualitative narratives of threat, contribution, authority, and alternative civic value, this pattern is consistent with the Homobiasos framework: collective reasoning may preserve moral coherence by making contradiction socially intelligible, absorbing change when it can be narrated as continuity and resisting it when it feels like assimilation or moral defeat. The Haredi case therefore offers both an empirical account of a community negotiating work, education, and civic obligation under pressure, and a broader theoretical lesson for polarized societies: durable integration is unlikely to emerge from correction, coercion, or ideological convergence alone; it is more likely when new obligations are both substantively real and morally inhabitable, allowing groups to expand participation without experiencing that expansion as the loss of their collective self.

## Data Availability

The datasets presented in this study can be found in online repositories. The names of the repository/repositories and accession number(s) can be found at: https://osf.io/3n6s8/overview.
