# Supplementary material for: Collective self-justification and resistance to integration: behavioral and social mechanisms within the Israeli ultra-orthodox community
Source: Front Sociol. 2026 Jul 13;11:1756939. doi: 10.3389/fsoc.2026.1756939 (PMC13403333; doi:10.3389/fsoc.2026.1756939)
Supplement: Supplementary file 1 [file Data_Sheet_1.DOCX]

Supplementary Material

# Supplementary Data

## Overview

The present report summarizes the main findings of the full Haredi Attitudes Survey, conducted in July 2025 during an ongoing military conflict and the national debate over the proposed conscription law. The survey sought to listen to voices from within a community, often spoken about yet rarely heard directly. The study aimed to provide empirically grounded insights into how Haredi individuals perceive key domains of everyday life, including education and employment, military service and civic duty, identity and belonging, and political attitudes, while trying to document the moral and psychological mechanisms underlying these perceptions.

# Methodology

## Sampling and Procedure

Data were collected in the last week of July 2025 through the Askaria Haredi Online Panel (https://askaria.co.il), a specialized survey company focusing on Israel’s ultra-Orthodox population. A total of N = 429 participants completed the questionnaire and received monetary compensation for their time. The sample included members of all major Haredi sub-streams—Lithuanian (22.9%), Hasidic (20.5%), Sephardic (45.5%), and Modern/Other (about 8%)—with demographic characteristics broadly consistent with national estimates for the Haredi population.

The survey was conducted online and included both closed-ended and open-ended questions. Despite the challenges of recruiting ultra-Orthodox participants for academic research, response rates exceeded 95%, suggesting high motivation to express views on socially significant issues.

*Questionnaire Structure*

The full questionnaire included 56 items, divided into the following sections:

• **Demographics** (15 items): gender, age, marital status, education, employment, political orientation, and religious stream.

• **Education and Employment** (9 items): attitudes toward professional training, higher education, and participation in the general labor market.

• **Conscription and Civic Duty** (10 items): opinions on military and civilian service, authority, and autonomy.

• **Identity and Belonging** (11 items): perceived conservatism, national pride, and communal identification.

• **Political Attitudes** (10 items): voting patterns and openness to a new “national Haredi” party.

• **Qualitative Question**: an open-ended prompt inviting participants to share thoughts or comments not covered by the structured items.

The full translated questionnaire appears in *Appendix A*.

*Analytic Approach*

Quantitative analyses included t-tests, ANOVAs, and Chi-square tests to examine group differences across gender and religious sub-streams. Given the large number of comparisons, statistical interpretations were made cautiously, emphasizing patterns with consistent directionality and high significance levels.

# Demographics

The final sample consisted of 429 Haredi participants, including 247 men (57.6%) and 182 women (42.4%), representing a relatively balanced gender composition compared to prior studies in this population. The mean age was 34.0 years (SD = 11.0), with ages ranging from the early twenties to the forties, indicating substantial representation of younger adults, who are most likely to be making key decisions regarding family, education, and employment.

A large majority (87.9%) were married, 10.9% were single, and the remainder were divorced or widowed. Participants reported an average of 4.1 children (range 0–12), somewhat below the national Haredi average (≈6.5), reflecting the relatively young age of respondents and the inclusion of Modern Haredi subgroups.

## Employment and Occupation

Employment patterns revealed the persistent tension between integration and religious commitment.

• 48.2% reported working in *non-religious (general)* occupations.

• 16.4% worked in *religious or communal* roles (e.g., teaching, outreach, or community service).

• 19.2% of men identified as *full-time yeshiva students (avrechim)*.

• Smaller proportions identified as *students (6.2%)*, *homemakers (2.1%)*, *retirees (0.7%)*, or *job seekers (2.9%)*.

Qualitative review of “Other” responses (2.6%) revealed hybrid cases such as *“running a private business while studying Torah”*, *“teaching in a secular kindergarten while studying,”* or *“living on disability allowance while learning part-time.”* These cases illustrate the adaptive strategies used to balance financial necessity and religious identity.

Overall, about two-thirds (≈65%) of respondents reported some level of labor-market participation, signaling gradual but cautious economic integration, driven mainly by practical rather than ideological motives.

## Political and Geographic Distribution

Voting patterns mirrored known communal alignments:

• 34.2% voted for *United Torah Judaism (UTJ)*,

• 44.3% for *Shas*,

• 8.4% abstained from voting, and

• The remainder supported other right-hand parties (*Likud, Otzma Yehudit,* etc.).

Participants came from a wide range of cities, with concentrations in Jerusalem (72), Bnei Brak (63), Beit Shemesh (40), Modi’in Illit (28), and additional representation from Ashdod, Elad, Netanya, Haifa, and Beitar Illit, covering both central and peripheral areas. This distribution reflects the geographic diversity of Israel’s Haredi society.

*Digital Access*

Digital connectivity has become increasingly salient for understanding social change within the Haredi community.

• 76.7% reported using the internet,

o of whom 55% used *filtered internet* (e.g., Netiv or Etrog),

o 13.7% used *light filtering* (e.g., Rimon), and

o 8% used *regular, unfiltered internet*.

• 23.3% reported no internet use at all.

• Regarding mobile phones, 60.5% used *kosher (non-smart)* phones, while the rest used smartphones, often with filtering apps.

These figures underscore the diversity within the Haredi population—from highly conservative groups maintaining traditional boundaries to others gradually integrating digital tools for work, education, and civic engagement.

# Attitudes Towards Higher Education and Livelihood

## General Attitudes Toward Work and Professional Training

Five items assessed participants’ views on economic independence, vocational education, and participation in the general labor market. Internal reliability was high (α = .73), allowing the computation of a composite index (M = 3.50, SD = 0.87, on a 1–5 scale). A one-sample t-test showed that support for integration into the workforce was significantly higher than the neutral midpoint (*t*(418) = 11.75, *p* < .001), indicating a generally positive—though not unqualified—attitude toward employment.

A one-way ANOVA revealed significant differences across sub-streams (*F*(3, 415) = 22.24, *p* < .001).

• Hasidic respondents expressed the highest support (M = 3.95, SD = 0.68).

• Modern/Other respondents also showed high endorsement (M = 3.83, SD = 0.77).

• Sephardic Haredim followed (M = 3.55, SD = 0.76).

• Lithuanian Haredim reported the lowest support (M = 3.18, SD = 0.90).

Gender differences were also significant, *F*(1, 417) = 11.87, *p* < .001, with men (M = 3.62, SD = 0.83) expressing stronger approval of work than women (M = 3.33, SD = 0.91). These results highlight meaningful variation within Haredi society, where Hasidic, Modern, and Sephardic groups show greater openness to work, while Lithuanian communities remain more reserved.

## Motivations for Education and Employment

When asked to identify the two main motivations for pursuing education or work (from six options), the dominant response—chosen by 79.7%—was economic necessity. The next most common motives were developing one’s abilities or career (29.6%), personal interest (22.8%), and problems in the current structure (16.8%). Only 9.8% mentioned the desire for broader social integration, and 4.4% selected “Other.”

Motivational patterns were broadly consistent across sub-streams, though the Lithuanian group stood out for citing difficulties within the yeshiva framework (23.8%) significantly more often than others (χ²(3) = 15.30, *p* < .01). These findings underscore that economic pressure, not

ideological change, drives most of the movement toward education and employment. Cultural concerns and fear of erosion of religious identity continue to temper the scope of integration.

## Balancing Torah and Livelihood

Respondents were asked to choose their preferred model for combining Torah study and employment. The most frequent answers were:

• “Other” (29.8%), mostly open-text responses advocating partial employment alongside sustained Torah study;

• “Part-time work alongside study” (27.5%);

• “Culturally adapted professions” (20%);

• “Study by day, work at night” (11.7%); and

• “Gradual integration by age” (9.6%).

No significant differences emerged between sub-streams (χ²(12) = 13.91, *p* = .31), suggesting broad agreement on maintaining a religiously compatible balance between learning and earning. Qualitative responses emphasized flexibility (“each according to his situation”), profession alignment (“teaching, rabbinic roles, Torah-related writing”), and a minority stance rejecting work altogether (“study only”).

Taken together, these results illustrate a pragmatic yet cautious negotiation between economic realities and the preservation of Torah-centered life—a discourse increasingly prevalent within Haredi communities.

## Attitudes Toward Core Curriculum (General Studies)

Participants rated agreement with the statement *“Core studies (math, English, sciences) should be included in Haredi schools.”* Responses showed strong opposition:

• 34.7% “strongly disagreed” and 18.6% “disagreed,”

• 21.4% were “unsure,” and only 23.8% “agreed” or “strongly agreed.”

The mean rating (M = 2.44, SD = 1.34, 1–5 scale) was significantly below the midpoint (*t*(420) = –8.63, *p* < .001), reflecting clear resistance to curricular integration. Differences across sub-streams were significant (*F*(3, 418) = 5.97, *p* < .001):

• Lithuanians showed the strongest opposition (M = 2.14, SD = 1.29),

• Hasidim and Sephardim were slightly more moderate (M ≈ 2.68, SD ≈ 1.3),

• Moderns/Others showed the lowest opposition (M = 2.72, SD = 1.4).

These data confirm that while economic pragmatism drives support for work, the educational sphere remains a symbolic boundary of identity, defended even by those open to professional training. Core studies are viewed not as an academic issue but as a cultural threat.

## Support for Higher Education for the Younger Generation

When asked, *“Would you send your children or relatives to academic studies?”*, responses reflected both gender and ideological patterns:

• 38.5% would send daughters only,

• 2.1% would send sons only,

• 22.4% would send both, and

• 35% would not send either.

This strong gender asymmetry mirrors the accepted cultural norm that women, not men, may enter academia to support the family while men remain in Torah study. Yet some respondents—mainly younger or Modern Haredim—endorsed broader inclusion.

Differences between streams were statistically significant (χ²(9) = 45.85, *p* < .001):

• Sephardic Haredim showed the highest openness (61.6% in favor only of women’s higher education).

• Hasidic and Lithuanian respondents were more restrictive (about 42% against) but allowed female participation (around 40%).

• Modern Haredim displayed the greatest overall flexibility (43.5% supporting both genders and only 30.4% opposition).

Similar to previous findings, these patterns suggest that higher education for women has become normatively accepted, whereas men’s academic participation remains ideologically sensitive.

# Conscription and Civic Duty

## Authority and Decision-Making

When asked *“who should decide whether a yeshiva student serves in the army”*, an overwhelming 81.8% of respondents selected the rabbi, while 13.3% chose the individual himself, and only 5.0% said the family. This pattern indicates that moral agency is largely externalized. Authority is vested in rabbinic and communal leadership rather than in personal autonomy.

Yet, significant variations appeared across subgroups (χ²(6) = 17.65, *p* = .007): Modern (72.3%) and Sephardic (79.1%) were less likely to assume responsibility to the rabbi than Hasidic (85.6%) and Lithuanian (83.3%). Similarly, Modern (19.1%) and Sephardic (17.4%) favored individual discretion more than Hasidic (8.2%) and Lithuanian (12.5%).

Economic status was also significantly associated with the results (χ²(10) = 35.42, *p* < .001). Higher incomes were associated with lower rabbinic authority and higher individual discretion. For example, respondents with an income higher than NIS 25,000 chose the rabbi only 60.0% of the time and the individual 33.3%. By contrast, people with no income chose the rabbi 87.0% of the time, and the individual only 4.3% of the time. Such differences may reflect gradual shifts in social confidence among less economically dependent and moderately integrated families. However, rabbinic legitimacy remains the dominant axis of moral authority in all streams and economic statuses.

## Enlistment Endorsement

A clear majority opposed endorsing military service even when adjusted to the Haredi needs:

• 67.1% opposed endorsement,

• 23.1% supported endorsement, to some extent,

• Less than 10% say they are not certain.

Sub-stream differences were highly significant (χ²(18) = *34.73*, *p* = .01):

• Lithuanians showed the strongest opposition (73.6%).

• Sephardim were slightly less resistant (68.9%).

• Hasidim demonstrated greater openness (59.7%).

• Modern Haredim showed the highest support (40.3%).

This gradient aligns with broader ideological segmentation: resistance remains normative, but pragmatic exceptions appear in more modernized or socioeconomically mobile circles. Analyzing the data based on economic status showed a supportive trend, but it was not statistically significant (χ²(30) = *23.50*, *p* = .79).

## Civic Responsibility and Normative Dissonance

When asked to state to what extent they agree with the statement*, “It is important that everyone, regardless of sector, contributes to their country,”* only 37.8% agreed to some extent. By contrast, 52.6% said they disagree. The remaining 9.7% were not sure. Similar results were found across all streams (χ²(18) = *25.87*, *p* = .10).

## Recommending Service to Relatives

Another question asked whether respondents would recommend a close family member to enlist in either military or civilian service. Around 55% said they wouldn’t recommend either, while only 14.0% said they would recommend both. Another 28.3% indicated they would recommend civilian service, and only 1.9% said they would recommend only military service. Thus, even among those who previously endorsed service in principle, personal endorsement remained extremely low, indicating that openness is more pragmatic than ideological, explaining why it does not easily translate into behavioral willingness.

The results point to two gaps. The first is between general endorsement in military service (about 23%) and willingness to recommend it to relatives (less than 16%). The second is between opposition to endorsing the service (almost 70%) and unwillingness to recommend it (55%). This might point to a collective-pragmatic reasoning, which recognizes the value of contribution but avoids transgressing social boundaries. In line with this, despite significant differences between sectors (χ²(9) = *44.52*, *p* < .001), even in the most modern stream, most said they would not recommend either.

## Preconditions for Haredi Service

To understand the main barriers to army service, respondents were asked to choose the two most important conditions that should be met in order to enable Haredi military service, from the following list:

1. Strict observance of kosher dietary laws;

2. A closed, all-Haredi framework;

3. Integration of Torah study during service;

4. Haredi commanding officers;

5. A designated Haredi military corps;

6. Adaptation of IDF values to Haredi values.

The results clearly reveal a preference for conditions that preserve religious and communal identity, not technical or logistical issues. The two most frequently selected conditions were:

• Alignment of IDF values with Haredi values (59.9%), and

• A closed Haredi framework (57.1%).

The next most important condition was strict observance of kashrut (26.8%), while integration of Torah study (14.2%), a designated Haredi corps (11.0%), and Haredi commanding officers (10.3%) were considered much less critical.

The identity threat—that young Haredim will return from the army less observant—is intensified by the perceived gap between policy rhetoric and the practical implementation of integration programs. This fear is amplified by deep mistrust toward state institutions and secular motives, and by concern over losing communal influence on what is seen as the most precious asset: the religious identity of the younger generation. At the halakhic and educational level, potential recruits are at a formative stage in life, when Torah study and religious identity are at their peak, making them especially vulnerable to outside influence. The prevailing concern is that they may enter the army as Haredim and return as secular, without parents or rabbis being able to intervene.

## The Principle of *Toratam Omanutam* (Torah as Vocation)

To assess how strongly the Haredi public equates Torah study with military service, respondents rated their agreement (1-7) with the statement: *“The principle of Torah as vocation is no less important than the national conscription law.”*

The results show an almost complete consensus.

• 72.3% expressed full agreement,

• 13.8% agreed, and

• 2.3% slightly agreed.

In total, nearly nine out of ten respondents viewed Torah study as an equally vital, and often superior, contribution to the state compared with military service.

The consensus was extremely high among all conservative streams:

• Hasidic: 89.6% agreement,

• Lithuanian: 89.2%,

• Sephardic: 90.7%.

Among the Modern stream, endorsement dropped to 78.9%. Opposition rates were low overall but somewhat higher among Lithuanians (9.9%) and Modern (9.6%) compared with Sephardim (8.0%) and Hasidim (5.2%). The differences were statistically significant (χ²(21) = 48.95, p < .001). These findings suggest that *Toratam Omanutam* is not merely a slogan but a foundational identity principle that external pressure alone cannot dislodge.

## Perceived Change in Haredi Attitudes Toward Conscription

To examine whether the social pressure and public criticism in Israel have influenced Haredi attitudes toward military service, participants were asked, *“Do you think the Haredi community’s attitude toward conscription has changed in recent times?”* Overall, 44.9% of respondents disagreed or strongly disagreed that any change had occurred, while 34.2% agreed or strongly agreed. An additional 19.6% were uncertain.

Significant variation emerged across religious subgroups (χ²(15) = 63.69, p < .001):

• Hasidic: about half perceived *no change*; 29% perceived *some change*.

• Lithuanian: about half perceived *no change;* 35.8% reported noticing a change.

• Sephardic: 41.4% saw *no change*; 29% perceived *some change*.

• Modern: only 23.1% saw *no change*, whereas 48.1% perceived a *change*.

These figures indicate that perceived change is uneven across Haredi society: it remains limited in conservative streams and more visible among modern or socially integrated groups.

## Confidence in Political Representation

When asked whether Haredi political representatives would succeed in passing a new conscription law that exempts yeshiva students from military service, only 23.5% of respondents answered positively. In comparison, 29.1% expected the law to fail. Nearly half (45.5%) said they were unsure. This indicates two extremes, with opposing levels of confidence, whereas most remain uncertain rather than overtly pessimistic.

Differences across streams were significant (χ²(9) = 31.39, p < .001):

• Hasidic: 16.5% believed the law would pass; 34.0% believed it would fail; 49.5% were unsure.

• Lithuanian: 22.2% believed it would pass; 32.6% believed it would fail; 44.0% were unsure.

• Sephardic: 31.0% believed it would pass; 18.4% believed it would fail; 49.4% were unsure.

• Modern: 22.9% believed it would pass; 25.0% believed it would fail; 36.5% were unsure.

Across all groups, skepticism outweighed confidence. However, Sephardic and Modern Haredim expressed relatively higher trust in the ability of their political representatives, while Hasidic and Lithuanian respondents showed greater doubt and ambivalence.

## Perceived Political Risk Following a Potential Conscription Law Failure

When asked whether the failure to pass a new conscription law would weaken the political power of Haredi parties, 47.3% of respondents think or are certain it would, 23.6% believe it would not, and 27.8% were unsure about the consequences.

Responses were also coded on a 1–5 scale (1 = “certainly will weaken,” 5 = “certainly will not”). The overall mean was M = 2.59, SD = 1.20, significantly below the midpoint (t(420) = –7.07, p < .001), indicating a mild but significant tendency to anticipate political damage. This pattern reflects awareness of the issue’s political sensitivity but not an expectation of a dramatic collapse in electoral support.

A one-way ANOVA revealed significant differences between streams (F(3, 417) = 2.63, p = .05):

• Sephardic Haredim: M = 2.28, SD = 1.15 — highest level of concern

• Modern: M = 2.51, SD = 1.18

• Hasidic: M = 2.67, SD = 1.21

• Lithuanian: M = 2.70, SD = 1.20

Post-hoc comparisons showed that Sephardic respondents differed significantly from all other groups (p < .04), expressing the strongest fear of loss of political power.

Consistent with this pattern, Shas voters (mainly Sephardic Haredim) showed higher concern (M = 2.35, SD = 1.14) than other voters (M = 2.65, SD = 1.21; t(419) = –2.07, p < .04), suggesting that expectations are highest for the largest and most politically assertive Haredi party. Perceived risk was also linked to satisfaction with party performance: respondents satisfied with their party’s conduct reported M = 3.15 (SD = 1.1), while dissatisfied voters averaged M = 2.26 (SD = 1.1; t(369) = 7.44, p < .001). Lower satisfaction thus corresponded with greater perceived political vulnerability.

Overall, respondents expressed moderate concern that political failure could weaken Haredi influence, but not to the point of perceived existential threat. The Sephardic subgroup’s elevated concern likely reflects stronger institutional trust and higher expectations from its political leadership, whereas Hasidic and Lithuanian respondents, already skeptical of state institutions, show emotional insulation, buffering potential disappointment through reduced

expectations. In this sense, reactions to potential policy failure appear less about political calculation and more about identity regulation: communities may sustain internal coherence by reframing loss as endurance rather than defeat.

## Rabbinic Authority as a Barrier to Compromise

Respondents were asked whether they believe rabbinic positions make compromise on the conscription issue impossible. Responses were nearly evenly split: 43.3% said rabbinic stances *do* constitute a barrier, while 54.5% said they *do not*. Significant differences emerged between streams (χ²(6) = 54.183, p < .001). Among the more conservative groups, the majority viewed rabbinic leadership as *not* an obstacle:

• Hasidic: 60.8%

• Lithuanian: 56.5%

• Sephardic: 52.9%

By contrast, among the Modern group, only 38.5% held this view, indicating greater readiness to attribute the deadlock to rabbinic rigidity. Thus, peripheral and modern subgroups, those more exposed to external cultural, educational, and occupational contexts, assigned more responsibility to rabbinic positions. Still, these results are unlikely to indicate a loss of rabbinic legitimacy, which remained very high among all respondents.

## Predictors of Support for Adapted Service: Random-Forest Analysis

Goal: Identify the strongest predictors of support/opposition to adapted military or civilian service among Haredi respondents, using a non-parametric model that captures non-linearities

and interactions. The predicted variable was the answer to the question “How likely are you to endorse tailored Haredi military service (1–7 scale: 1 = “strongly oppose” … 7 = “strongly support”).

Data preparation (brief):

• Removed open-ended fields; retained 75 structured predictors.

• Dropped rows missing the outcome; dummy-coded categoricals; median imputation for predictors; removed duplicative variables.

• Train/test split: 80/20.

Model performance:

• Full model: 𝑅𝑅2≈0.58; RMSE ≈ 1.28.

• Top-11 predictors only: 𝑅𝑅2≈0.55(≈3% drop); RMSE ≈ 1.33.

Thus, adding more than 11 predictors will not significantly improve predictive power.

Variable Importance (top signals):

*Normative/attitudinal anchors*

• Willingness to recommend military or civilian national service to a close family member (especially both) is highly and positively associated with endorsing military service for Haredim.

• Agreement that everyone should contribute to the state (via army or national/civilian service) is also highly associated.

*(Consistent with planned behavior logic: attitudes & perceived norms align with intentions.)*

*Deference to religious authority*

• Agreement with *“I will not vote against my rabbi’s view”* and assigning enlistment decisions to rabbis are associated with lower endorsement. *(Collective authority cues temper individual openness to compromise.)*

*Integration indicators*

• Encouraging academic studies (men and women), support for core studies, and exposure to general media are positively associated with endorsement. *(Greater contact with general institutions reduces perceived identity conflict.)*

Political openness

• Openness to a new liberal/ national-Haredi party is positively associated with endorsement.

*Demographics (limited role)*

• Younger respondents are slightly more likely to endorse military service.

• Less conservative or religious jobs were associated with higher endorsement.

No explanatory power was found for stream, gender, income, and other “classic” demographics.

Model-based Psychological Profiles

• Supporters: Younger; higher general-media exposure; endorse academic studies; value contribution; comfortable with Haredi-compatible integration.

• Opponents: Older; strong rabbinic deference; oppose academic studies and professionalization; low external media exposure.

• Undecided: Mixed signals—Acknowledge the required changes, but assume responsibility to protect the collective; some pragmatic exposure (e.g., work) without major attitudinal shift.

# Identity and Belonging

## Self-Placement: Conservatism vs. Modernity

Respondents located themselves on a 5-point scale from “very conservative” to “very modern,” with a middle “both/and” category. The distribution indicates a non-binary identity profile: while more than half (52.0%) identified as conservative/very conservative, 38.2% chose both/and (the most frequent category), and less than 9% chose modern (7.7%) or very modern (0.7%). Thus, beyond a conservative majority, a sizable minority reports a hybrid positioning rather than a sharp dichotomy.

Significant differences were found between streams (χ²(6) = 23.231, p < .001). Among Lithuanians, 63.5% chose conservative and 30.2% both/and, with only 6.3% modern. Among Hasidim, 53.4% conservative, 37.3% both/and, and 9.3% modern. Among Sephardim, 48.3% conservative, 49.4% both/and, and 2.3% modern. Finally, in the Modern group, only 36.2% conservative, 42.5% both/and, and 21.3% modern. Overall, identity self-definitions track communal contexts and institutional boundaries, with both/and signaling a prevalent hybrid identity, consistent with recent findings.

## Importance of Community Belonging

When asked how important it is for them to belong to their community, nearly 90% rated it very (41.5%) or quite (45.0%) important. By contrast, only 10.0% rated it quite unimportant, and 2.1% not important at all. Stream differences were significant (χ²(3) = 10.090, p < .02), with Hasidim (96.9%) rating it highest, followed by Lithuanians (85.4%) and Sephardim (85.1%), with Modern exhibiting the lowest importance (83.0%). Despite these significant differences,

belonging to the community is very important to all streams, aligning with known variations in communal cohesion intensity.

## Is Haredi Society Becoming More Conservative or More Open?

Perceived direction of change shows broad dispersion: 11.2% said their community becomes much more Haredi, and 13.1% said somewhat more Haredi. Thus, a little less than 25% believe the Haredi community is becoming more conservative. By contrast, 46.9% believed their community was somewhat more open, and 8.2% said it was much more open. Thus, most (≈55%) identify their society as becoming more modern. The remaining 19.1% see no substantial change.

A main significant effect was found for stream (χ²(6) = 25.343, p < .001). Modern (74.5%) and Sephardim (61.6%) most often perceived growing openness; Lithuanians (56.3%) were more moderate, while Hasidim showed the lowest perceived change towards moderna (41.2%). Similarly, Hasidim showed the highest share of those perceiving greater conservatism (40.2%), followed by Lithuanians (21.9%), Moderns (19.1%), and Sephardim (16.3%). “No change” clustered around ~18–22% in conservative streams and 6.3% among Moderns. These patterns match differences observed in employment, education, and attitudes toward conscription.

Importantly, the gap between self-placement, which locates most individuals on the conservative side of the continuum, and perceived change, which suggests the community is becoming more modern, provides an important insight. While group members identify changes in livelihood and higher education as necessities at the collective level, they do not internalize

this pragmatic shift, accommodating the apparent change towards modernity without risking their personal Haredi Identity.

## Decision Influencers in Key Life Domains

Respondents indicated how often they consult various sources (1 “not at all” to 5 “very much”) for major decisions (family, education, politics, conscription, etc.):

• Rabbi: M = 3.76, SD = 1.19; above midpoint (t(418) = 12.978, p < .001). The primary reference across streams.

• Family: M = 3.48, SD = 1.53; above midpoint (t(405) = 7.941, p < .001). Family is a central arena of influence.

• Admore (Hasidic Rebbe): M = 2.04, SD = 1.53; below midpoint (t(405) = −12.617, p < .001). As expected, relevance is stream-specific and aligns with charismatic leadership patterns.

• Community friends: M = 2.78, SD = 1.31; below midpoint (t(401) = −3.349, p < .001).

• Professional advisors: M = 2.91, SD = 1.32; not different from midpoint (t(403) = −1.434, p = .152). Highest among Modern (3.11), lowest among Lithuanians (2.73).

• Mass media: Lowest influence, M = 1.43, SD = 0.80; below midpoint (t(399) = −39.434, p < .001); no significant stream differences.

Overall, authority is hierarchical: rabbinic guidance first, family second, community third, external sources last.

## Sense of Belonging to the State of Israel

Respondents were asked, *“How much do you feel part of the State of Israel and its problems?”* (1–5). Overall, the mean score across streams indicated a moderate but significant sense of belonging (M = 3.42, SD = 1.27), above the midpoint (t(417) = 6.809, p < .001). Significant differences were also found between streams (F(3, 414) = 9.045, p < .001): Sephardim reported the highest sense of national belonging (M = 3.91, SD = 1.11), then Hasidim (3.56, SD = 1.19) and Modern (3.53, SD = 1.25); Lithuanian felt the least sense of belonging (3.11, SD = 1.31), yet still above the midpoint.

Belonging at the personal level was significantly correlated with attitudes towards army service endorsement (r = 0.312) and agreement that everyone should contribute to the state (r = 0.39; all p < .001). Partial correlations within streams were nearly identical, indicating that these associations are similar across streams. Belonging relates more to the general principle of communal responsibility than to the more specific, identity-threatening behavioral requirement.

## Pride in Being Israeli

Agreement with “I am proud to be Israeli” (1–5) also yielded a moderate mean: M = 3.26, SD = 1.36; above the midpoint (t(419) = 3.996, p < .001). Similar trends to belonging were found among streams (F(3,416) = 11.977, p < .001): Sephardim highest (M = 3.92, SD = 1.12), Modern (3.36, SD = 1.47), and Hasidim (3.32, SD = 1.28) intermediate, Lithuanians lowest (2.92, SD = 1.35).

Sense of pride at the personal level was highly correlated with belonging (r = 0.65, p < 0.001). Moderate significant correlations were also found with endorsement of attitudes towards army service (r = 0.33) and agreement that everyone should contribute to the state (r = 0.41; all p < .001). Partial correlations within streams were nearly identical. Like belonging, pride too

relates more to the general principle of communal responsibility than to the more specific, identity-threatening behavioral requirement.

# Political Attitudes

## Satisfaction with My Political Party

Participants rated their level of satisfaction with the party they voted for in the last election on a 1–5 scale (“very satisfied” to “disappointed”). The overall mean was 3.02 (SD = 1.22). Because three of the five response options express some level of dissatisfaction, a mean of 3 indicates mildly negative satisfaction rather than neutrality. A one-sample t-test confirmed the mean is significantly lower than 4, the positive benchmark (t(373) = –15.644, p < .001). To account for

scale asymmetry, we also computed a binary indicator (“satisfied”/“very satisfied”): only 38.5% reported positive satisfaction, and over 60% were not satisfied or felt their expectations were unmet.

Small differences in streams – Hasidim (2.80, SD = 1.30), Lithuanian (3.00, SD = 1.22), Sephardic (3.18, SD = 1.1), and Modern (3.22, SD = 1.00) – were not statistically significant (F(3, 370) = 1.755, p = .155). However, a significant effect was found for vote choice, with non-Haredi party voters (mainly Otzma Yehudit and Likud) reporting the highest satisfaction (M = 3.30, SD = 1.34), then Shas voters (M = 3.24, SD = 1.15), and United Torah Judaism (UTJ) voters the lowest (M = 2.91, SD = 1.21).

## General Political Items

Four different items (1-5 agreement scale) were used to examine general political attitudes.

1. Personal Representation: *“Haredi parties represent my needs”*

Mean agreement was 2.64 (SD = 1.27), significantly below the scale midpoint (t(423) = –5.90, p < .001), indicating mild disagreement. By stream, Lithuanian respondents had the lowest disagreement (M = 2.74, SD = 1.37), followed by Sephardic (M = 2.67, SD = 1.15). Modern (M = 2.49, SD = 1.11) and Hasidim (M = 2.48, SD = 1.20) had the highest disagreement levels. However, these differences were not significant (F(3, 420) = 1.083, p = .356). By voting, non-voters reported the highest disagreement (M = 1.67, SD = 0.95), followed by non-Haredi voters (M = 2.25, SD = 1.25). Next were Shas voters (M = 2.75, SD = 1.13), and finally UTJ voters, who reported the lowest disagreement (M = 2.82, SD = 1.28); differences were significant (F(3, 420) = 13.542, p < .001).

2. Deference to Rabbinic Authority: *“I will not vote against my rabbi’s opinion”*

Mean agreement was high (M = 4.26, SD = 1.16). By stream, Hasidim (M = 4.42, SD = 1.04) and Lithuanians (M = 4.37, SD = 1.10) were higher than Sephardi (M = 3.99, SD = 1.30) and Modern (M = 4.02, SD = 1.29). These differences are significant (F(3, 419) = 4.564, p < .02). By voting, UTJ voters were highest (M = 4.46, SD = 0.96), followed by Shas (M = 4.07, SD = 1.22) and non-voters (M = 4.04, SD = 1.49); non-Haredi voters were lowest (M = 3.37, SD = 1.52), F(3, 419) = 10.448, p < .001.

Using Spearman correlations between general reliance on one’s rabbi for important decisions and domain-specific items (FDR-adjusted q values), the strongest associations clustered around conscription and core-curriculum education:

• Conscription: higher reliance associated with preference that the rabbi decide enlistment (rₛ = –.39, p < .001, q < .001), lower endorsement in support for tailored military service (rₛ = –.29, p < .001, q < .001), and lower valuation of national service/contribution (rₛ = –.22, p < .001, q < .001).

• Core studies and higher education: lower support for core studies (rₛ = –.36, p < .001, q < .001) and for sending children to academic studies (rₛ = –.28, p < .001, q < .001).

• Politics: positive association with agreeing not to vote against the rabbi (rₛ = .33, p < .001, q < .001), but no link with expecting communal sanctions against dissenters (rₛ = .01, p = .87, q = .87).

• Labor and economy: weak/limited links—slightly lower support for encouraging labor force participation (rₛ = –.28, p < .001, q < .001), and no association with valuing economic independence (rₛ = .03, p = .58, q = .64).

• Family/community: small positive link with consulting the community (rₛ = .15, p = .003, q = .005), none with consulting family (rₛ = .09, p = .066, q = .081).

Taken together, the results suggest that rabbinic influence focuses more on identity-boundary issues (e.g., conscription, core curricula) than on pragmatic domains (e.g., work, household decisions).

3. Preference for Choice in the Haredi Public Sphere: *“It is important to allow more choice within Haredi society.”*

This item was reverse-coded so that higher scores indicate greater conservatism. The mean was 2.80 (SD = 1.31), significantly below the midpoint (t(421) = –3.120, p = .002). This might suggest a small but significant preference toward accommodating greater individual choice in politics.

When analyzed by stream, Lithuanians were most resistant (M = 3.02, SD = 1.32), Hasidim next (M = 2.74, SD = 1.35), while Sephardi (M = 2.56, SD = 1.25) and Modern (M = 2.48, SD = 1.17) were more supportive (F(3, 418) = 6.319, p = .011). By voting, non-voters (M = 3.02, SD = 1.27) and UTJ voters (M = 2.95, SD = 1.33) were most resistant; Shas voters less so (M = 2.45, SD = 1.24); non-Haredi voters most supportive (M = 2.13, SD = 0.97; F(3, 418) = 10.555, p < .001).

4. Anticipated Social Sanction: *The Haredi community will reject those who vote against the rabbi.”*

The mean score was 3.14 (SD = 1.16), slightly but significantly above the midpoint (t(419) = 2.525, p < .02). This finding indicates a weak expectation of social pushback. By stream, Lithuanians were highest (M = 3.33, SD = 1.16), followed by Sephardic (M = 3.05, SD = 1.17) and Hasidic (M = 3.01, SD = 1.13). Modern had the lowest agreement level (M = 2.83, SD = 1.08). These differences were significant (F(3, 416) = 3.425, p < .02). By voting, UTJ voters reported the highest agreement (M = 3.27, SD = 1.12); non-Haredi (M = 2.97, SD = 1.25) and Shas (M = 2.94, SD = 1.10) voters had lower agreement, and non-voters had the lowest (M = 2.89, SD = 1.33; F(3, 416) = 2.907, p < .04).

Across four items, a consistent pattern emerges. Satisfaction with party performance and perceived representation are modest to low. Deference to rabbinic authority remains high, focused especially on identity-defining arenas (conscription, curricula), with weaker reach into day-to-day economic/family spheres. There is a small but significant opening toward greater individual choice in areas not considered to risk collective identity, which is more pronounced among Sephardic and Modern, and among those who cross partisan lines. Expectations of social sanction exist but are weak on average and vary by stream and partisan alignment.

Sephardi respondents occupy a nuanced middle: relatively greater openness to individual choice and somewhat lower rabbinic dependence than Lithuanian/Hasidic groups, yet with conscription attitudes similar to those of the broader Haredi public. This suggests a boundary-management strategy that balances belonging with selective autonomy. Sustaining communal affiliation while exercising personal discretion in education, work, and some political judgments.

# Potential for a National-Haredi Alignment

## Openness in Principle

Participants rated agreement with the statement *“There is room for a new national-Haredi party”* (1–5). The overall mean was M = 2.33, SD = 1.36, significantly below the neutral midpoint (t(417) = −10.126, p < .001), indicating overall mild resistance. A one-way ANOVA showed significant stream differences (F(3,414) = 3.89, p = .009): Lithuanian respondents were most opposed (M = 2.11, SD = 1.35), followed by Hasidim (M = 2.38, SD = 1.34); Sephardic (M = 2.52, SD = 1.29) and Modern (M = 2.76, SD = 1.46) were relatively less resistant.

By vote choice, differences were larger (F(3,414) = 9.952, p < .001): non-voters were most opposed (M = 1.89, SD = 1.33), with lower opposition among UTJ (M = 2.21, SD = 1.33) and Shas (M = 2.52, SD = 1.23) voters. Haredim who voted for non-Haredi parties were the only group with modest openness (M = 3.43, SD = 1.48).

## Would You Consider Voting for Such a Party?

When framed concretely, *“If a liberal national-Haredi party promoted employment, education, conscription, and equality, would you consider voting for it?”*—responses were markedly negative: 53.8% “certainly not,” 17.0% “probably not,” 17.0% undecided, 7.5% “probably yes,”

and 3.3% “certainly yes.” On a 1–5 scale, the mean was M = 1.88, SD = 1.14, significantly below midpoint (t(422) = −20.201, p < .001). Stream differences remained significant (F(3,419) = 4.374, p = .005): Lithuanian (M = 1.71, SD = 1.07) and Hasidic (M = 1.80, SD = 1.11) were most opposed; Sephardic (M = 2.15, SD = 1.20) and Modern (M = 2.19, SD = 1.28) were less so, yet still negative on average. By vote choice (F(3,419) = 11.318, p < .001): non-voters (M = 1.42, SD = 0.99) and UTJ (M = 1.76, SD = 1.05) were most resistant; Shas (M = 2.21, SD = 1.18) and non-Haredi-party voters (M = 2.67, SD = 1.42) were relatively more open.

## Softening the Platform: Removing Conscription

A “softened” scenario removed conscription from the platform (*“…promoted employment, education, and equality, but not conscription”*). The mean increased to M = 2.26, SD = 1.50, still below the midpoint (t(322) = −8.908, p < .001). Within-person differences (with vs. without conscription) showed a significant shift toward consideration (ΔM = −0.57, SD = 1.13; negative values = more openness; t(321) = −9.035, p < .001). However, the shift did not differ across streams (F(3, 318) = 1.267, p = .286). Conscription, thus, seems to function as a symbolic “red line.” Removing it reliably eases resistance, but only to a limited extent and for a minority.

## Perceived Community Willingness (Pluralistic Norms)

Respondents were asked how many of their Haredi friends would consider voting for such a party. Here too, the consensus was against high quantities: 20.5% answered none, and 48.5% said only a small minority. By contrast, 22.6% said several, 5.1% said many, and only 1.6% said most of their friends would consider voting for such a party. Analyzing by stream yielded a significant association (χ²(3) = 7.693, p = .05): Lithuanian (74.6%) and Hasidic (70.1%)

respondents most often predicted near-zero community willingness, while Sephardic and Modern were less pessimistic (~60%).

Personal willingness correlated only moderately with perceived peer willingness (ρ = .45, p < .001). Notably, 46 respondents (~10.9%) were “isolated supporters”: personally open, yet estimating that none of their friends would be. This aligns with pluralistic ignorance—privately held openness masked by expectations of communal rejection.

## What Would Increase Support?

Respondents prioritized rabbinic legitimacy (63.2%), commitment to Torah values (38.0%), a Haredi leader at the helm (33.1%), and family-oriented benefits (33.3%) as considerations that could sway them. Support from other Haredim was rarely decisive (10.0%), with 14.0% selecting “Other.” Only rabbinic legitimacy varied by stream (χ²(3) = 12.106, p = .007): highest among Lithuanian (74.6%) and Hasidic (70.1%); lower among Sephardic (60.9%) and Modern (59.6%).

Open-ended “Other” responses overwhelmingly reiterated rabbinic authorization (often naming specific rebbes), underscoring authority as a non-negotiable gatekeeper. Only 7.5% of the responses included consideration not directly related to rabbinic or divine authority.

## Main Obstacles

Respondents were also asked about the main barriers to such a party (multiple choice). The leading obstacle was lack of rabbinic support (77.2%)—even higher than the share listing rabbinic endorsement as a positive requirement—followed by fear of identity loss (37.3%) and distrust in politicians (33.8%). Community pressure (23.8%) and low awareness of this political option (13.3%) were cited less frequently; 6.3% chose “Other.”

Qualitative comments pointed either to entrenched power structures (“the public is captive to lobbyists,” “will not get enough votes”) or to fears of secularization if such a project gained traction.

# Qualitative analysis of the open-ended question

At the end of the survey, participants were invited to write freely about any thoughts, feelings, or general remarks they wished to share, without thematic or length restrictions. Over one hundred textual responses were received, of which approximately 65 were deemed relevant and substantive. A qualitative content analysis identified six recurring themes that illuminate the emotional and cognitive complexity of Haredi reasoning regarding state, identity, and service.

## Theme 1: The Army as a Melting Pot Threatening Haredi Identity

Many respondents repeatedly described the army not merely as a security institution but as an ideological space whose implicit purpose is to reshape religious identity. “The army contradicts halakha,” wrote one participant, and another added, “a person who follows orders becomes a slave—something impossible according to Jewish law.” Others referred to the IDF as “a melting pot,” “the army of the secular state,” or even “the army of those who sacrifice themselves for Israel.” In this framing, military service signifies participation in a system designed to erase the Haredi self and turn the Haredim secular.

## Theme 2: Pride and Persecution

Alongside opposition, many respondents expressed profound pride in their Haredi identity, often framing it as a spiritual mission. “I am proud to be among the persecuted and not among the persecutors,” one wrote, another declared, “The Haredi public contributes the most in spirit and economy.” Such expressions reveal a self-concept of moral superiority to mundane secular contributions. This dual narrative—of pride through victimhood—strengthens internal solidarity while morally justifying separation and nonparticipation in state service.

## Theme 3: Justifying Nonparticipation—Toward Others and Toward the Self

A notable pattern involved attempts to justify non-enlistment or nonintegration, not only as a public stance but also as an internal coping mechanism, reflecting dissonance between belonging and disengagement. As several wrote: “We volunteer in Magen David Adom, in ZAKA, in United Hatzalah—that is our contribution,” or “The Haredim contribute the most—in prayer, in spirit, and in deeds.” This reflects a moral rationalization mechanism: respondents reframe the meaning of contribution so that spiritual and communal acts outweigh civic duties. Consequently, accusations of “draft-dodging” or demands for “equal burden” are perceived as

delegitimizing and even reinforce withdrawal. One respondent quoted his rabbi: “He told us not to enlist, because they curse us even when we serve; we serve the state in a higher way.”

## Theme 4: Between Forced Enlistment and Religious Coercion

Some comments revealed striking internal contradictions that epitomize the moral dissonance within Haredi society. Participants who fiercely opposed coercive enlistment or “secular imposition” simultaneously endorsed “complete submission to Torah authority” and rejected individual choice. Freedom, in this sense, is applied selectively: one may resist external coercion while accepting absolute internal control. As one pattern suggests, while many reject the idea of state coercion in drafting yeshiva students, they find no issue with enforcing religious observance, such as Sabbath laws. This asymmetry indicates that the guiding principle is not universal freedom but loyalty to sectoral identity sustained by dichotomous reasoning.

## Theme 5: Intragroup Ambivalence and Layered Identities

Reflecting the broader ideological diversification of the Haredi world, some responses conveyed ambivalence and complexity. Participants described themselves as torn between conflicting yet complementary identities—Haredi, Israeli, and familial. “The Haredi society is opening on one side and becoming more extreme on the other,” one noted; another added, “The more one side pulls, the more the other side drifts to the opposite extreme.” These voices attest to an internal ideological split and to an ongoing redefinition of what counts as legitimate within Haredi boundaries. Rather than signaling disintegration, such ambivalence may reflect adaptive negotiation—an evolving equilibrium between competing loyalties.

## Theme 6: Deference to Rabbinic Authority as Moral Shield

Many respondents described absolute reliance on rabbinic authority: “The decision belongs to the rabbi alone,” “We have nothing to do with the state; we only follow God’s word,” or even, “We do not trust our own limited intellect.” Such statements express not only loyalty but also psychological relief from moral responsibility—a protective mechanism that reduces the anxiety of moral conflict in a changing reality. Some explicitly admitted the dissonance: “This questionnaire deals with issues only rabbis should decide,” or “It raises unpleasant feelings about the rabbi’s role in my life.” Deference thus operates both as a social norm and as a coping device that preserves coherence amid tension between obedience and autonomy.

# Limitations

Alongside its empirical contribution, the survey has several methodological and contextual limitations that should be acknowledged when interpreting and generalizing its findings.

*Sampling limitations* The survey relied on a specialized online panel of ultra-Orthodox participants. Although the panel is dedicated to this population, it naturally represents individuals with internet access, even when filtered through strict supervision. Consequently, the most insular groups—those who avoid digital communication altogether—are likely underrepresented. This underrepresentation may slightly bias the results toward more open attitudes, particularly regarding employment, higher education, or military service. In addition, the overrepresentation of the Lithuanian stream and the relative underrepresentation of some Hasidic and Modern subgroups may limit the ability to infer conclusions about the Haredi population as a whole.

*Selection bias* The exceptionally high response rate (above 95%) strengthens data reliability but may also

suggest self-selection by respondents who are more engaged in public discourse or more motivated to express and justify their views. Those with strong opinions, either defensive or reform-oriented—may therefore be overrepresented, while more indifferent or extreme voices could be less visible.

*Internal representativeness* Although the sample was stratified by stream, gender, age, and income, it may not capture the full diversity of the ultra-Orthodox world, including small, peripheral, or marginal communities. Furthermore, for analytical purposes, several smaller groups (e.g., Modern, Chabad, and Breslov) were merged into a single category, a necessary simplification that likely obscures finer distinctions among subgroups.

*Questionnaire design and self-report* The survey relied primarily on self-report measures, which are inherently sensitive to social desirability bias. Respondents may have preferred to appear “moderate” or “balanced,” especially on socially or politically charged topics such as conscription, core studies, or academic integration. Even carefully neutral phrasing can influence responses; for example, framing a statement as “encouragement” rather than “obligation” may yield systematically different patterns of agreement.

*Statistical and analytical limitations* Given the large number of between-group comparisons, the risk of Type I error (spurious statistical significance) cannot be fully eliminated, despite corrective procedures. Moreover, some statistically significant differences were small in magnitude and may not hold practical significance. Accordingly, interpretations should focus on consistent patterns rather than isolated effects.

*Temporal and contextual limitations* The survey was conducted in late July 2025, during a politically and socially charged period marked by ongoing national debates and security events. Haredi attitudes may have been influenced by short-term contextual factors—such as parliamentary disputes over the conscription law or episodes of national crisis—so some findings may reflect transient sentiment rather than long-term structural change.

*Interpretive implications* These limitations do not diminish the survey’s contribution but call for interpretive caution. The findings provide a rich, up-to-date portrait of a large and meaningful segment of Haredi society, yet they do not represent every subgroup or temporal context. Some discrepancies between personal and normative attitudes, or between streams, might sharpen or fade under different conditions or with alternative methodologies.

# Acknowledgements

I am deeply grateful to **Prof. Nissim Leon** and **Dr. Tehila Kalagy** for their insightful comments on an earlier version of this report and for affirming the importance and validity of its findings.

Special thanks are due to **Eli Shimoni, CPA**, whose inspiration and assistance in formulating the survey questions were instrumental in shaping this project.

Above all, I extend my heartfelt gratitude to the **429 participants** who opened their homes and hearts—with honesty and courage—offering a rare and moving window into the inner world of Haredi society in Israel. Their willingness to share their experiences, reflections, and personal perspectives made this work possible.

# References

Ajzen, I. (1991). The theory of planned behavior. Organizational Behavior and Human Decision Processes, 50(2), 179–211. https://doi.org/10.1016/0749-5978(91)90020-T

Ajzen, I. (2020). *The theory of planned behavior: Frequently asked questions*. *Human Behavior and Emerging Technologies, 2*(4), 314–324. https://doi.org/10.1002/hbe2.195

Ami, I. B. (2022). Modern Haredim and contemporary Haredi society: Beyond the paradigm of liberalization. *Jewish Social Studies, 27(2)*, 183-205.

Ben-Gurion, D. (1947, June 19). Letter to the Agudath Israel leadership regarding religion and state arrangements. Israel State Archives.

Blumen, O. (2002). Criss-crossing boundaries: Ultraorthodox Jewish women go to work. *Gender, Place and Culture: A Journal of Feminist Geography, 9(2)*, 133-151.

Brown, B. (2023). The Fundamental Components of Haredi ideology (Hashkafah). In K. Caplan

& N. Leon (Eds.), *Contemporary Israeli Haredi Society* (pp. 32-66). Routledge.

Cahaner, L. (2023). The study of Haredi space in Israel: Trends, characteristics, achievements, and challenges. *Contemporary Israeli Haredi Society*, 125-159.

Cahaner, L., & Malchi, A. (2022). Haredi labor market integration policy in a neoliberal environment. *Journal of Israeli History, 40(1)*, 137-159.

Campbell, H. A., & Tsuria, R. (2021). *Digital religion: Understanding religious practice in digital media*. Routledge.

Caplan, K., & Leon, N. (2024). *Contemporary Israeli haredi society*. Routledge.

Cohen, Y., Adini, B., & Spitz, A. (2021). The Haredi media, religious identity, and the COVID 19 crisis. *Israel Affairs, 27(5)*, 921-935.

Cooper, L. (2024). Hasidim praying for IDF soldiers after October 7 2023. *Contemporary Jewry, 44(4)*, 847-883.

Ellemers, N. (2017). *Morality and the regulation of social behavior: Groups as moral anchors*. Routledge.

El-Or, T. (2002). *Next year I will know more: Literacy and identity among young Orthodox women in Israel*. Wayne State University Press.

Feldman, A. (2019). Education and employment among ultra-orthodox women in Israel: modernity and conservatism—the case of the shas party. *Contemporary Jewry, 39(3)*, 451-472.

Finkelman, Y. (2011). Ultra-orthodox/Haredi education. In *International handbook of Jewish education* (pp. 1063-1080). Dordrecht: Springer Netherlands.

Finkelstein, A. (2024). The Political Structure of Haredi Local Authorities and Its Influence on How They Operate. The Israel Democracy Institute. https://en.idi.org.il/articles/52891

Friedman, M. (1991). The Haredi (ultra-orthodox) society: Sources, trends and processes. *Jerusalem: The Jerusalem Institute for Israel Studies, 1*, 80-87.

Gross, J. J., Halperin, E., & Porat, R. (2013). Emotion regulation in intractable conflicts. *Current Directions in Psychological Science, 22(6)*, 423-429.

Haidt, J. (2001). The emotional dog and its rational tail: A social intuitionist approach to moral judgment. *Psychological Review, 108*(4), 814–834.

Hochman, G. (2024). Beyond the Surface: A New Perspective on Dual-System Theories in Decision-Making. *Behavioral Sciences, 14*, 1028.

Hochman, G. (2025). Rationalization as cognitive homeostasis: A Homobiasos theory of adaptive self-regulation. Target article proposal invited for Behavioral and Brain Sciences (BBS). *PsyArXiv*. https://doi.org/10.31234/osf.io/w5cqr_v1

Hochman, G., Kalagy, T., Malul, S., & Yosef, R. (2025). Choosing not to know: The emotional and sociocultural architecture of pension willful ignorance. *Current Opinion in Psychology*, 102181.

Hochman, G., Peleg, D., Ariely, D., & Ayal, S. (2021). Robin Hood meets Pinocchio: Justifications increase cheating behavior but decrease physiological tension. *Journal of Behavioral and Experimental Economics, 92*, 101699. https://doi.org/10.1016/j.socec.2021.101699

Hochman, G., Yosef, R., Malul, S., & Kalagy, T. (2024). Escape from retirement: Characterizing

the barriers to pension information in the Israeli public. *Social Security, 123*, 77-113.

Israel Democracy Institute. (2024). *The Israeli Democracy Index 2024: Public opinion on democracy, trust, and polarization*. Jerusalem: Israel Democracy Institute. Retrieved from https://en.idi.org.il/media/27509/israel-democracy-index-2024-e_for_web_17-2-2025.pdf

Kahan, D. M. (2013). Ideology, motivated reasoning, and cognitive reflection. *Judgment and Decision making, 8(4)*, 407-424.

Kahneman, D., & Tversky, A. (1979). Prospect theory: An analysis of decision under risk. *Econometrica, 47*(2), 263–291. https://doi.org/10.2307/1914185

Kalagy, T., & Braun-Lewensohn, O. (2019). Agency of preservation or change: Ultra-Orthodox educated women in the field of employment. Community, *Work & Family, 22(2)*, 229-250.

Keren-Kratz, M. (2025). The Turnaround in Israel’s Haredi Society in the Late 20th Century: A Data-Based Analysis. *Religions, 16(2)*, 145.

Kunda, Z. (1990). The case for motivated reasoning*. Psychological Bulletin, 108*(3), 480–498.

Leach, C. W., Ellemers, N., & Barreto, M. (2007). Group virtue: the importance of morality (vs. competence and sociability) in the positive evaluation of in-groups. *Journal of Personality and Social Psychology, 93(2)*, 234.

Leon, N. (2016). The Haredi-secular debate and the Shas approach. *Handbook of Israel: Major Debates*. Berlin, De Gruyter Oldenbourg, 131-145.

Leon, N. (2020). A different hue of blackness: The Haredi case. In U. Dorchin & G. Djerrahian (Eds.), *Blackness in Israel* (pp. 130-143). Routledge.

Leon, N. (2023). Soft Ultra-Orthodoxy: Revival Movement Activists, Synagogue Communities and the Mizrahi-Haredi Teshuva Movement in Israel. *Religions, 14(1)*, 89.

Leon, N. (2024). “The Day After”—Initial Reactions of Haredi Society to the Israel-Hamas War. *Israel Studies, 29(1)*, 122-134.

Lerner, H. (2009). Entrenching the status quo: Religion and the constitution-making process in Israel. *Constellations, 16*(2), 306–329. https://doi.org/10.1111/j.1467-8675.2009.00538.x

Malach, G., & Cahaner, L. (2023). *The yearbook of ultra-Orthodox society in Israel 2023*. The Israel Democracy Institute. https://en.idi.org.il/publications/49123

Malchi, A. (2021). *Outsiders in Uniform: From the Margins to the Army and Back*. Jerusalem: Israel Democracy Institute. https://www.idi.org.il/media/16409/outsiders-in-uniform-from-the-margins-to-the-army-and-back.pdf

Malovicki-Yaffe, N., Itzhaki-Braun, Y., & Shahar-Rosenblum, S. (2023). Enlisting in the army in the Jewish Ultraorthodox community and the consequences for wellbeing. *Frontiers in Psychology, 14*, 1132624.

Neriya-Ben Shahar, R., Yuval, F., & Tur-Sinai, A. (2024). “I would consult a doctor, but what the rabbi says goes”: Ultra-orthodox Jews’ relationships with rabbis and doctors in Israel. *Journal of Religion and Health, 63(3)*, 1905-1933.

Perelman, Y., Goldberg, C., & Yaish, M. (2024). What Drives Ultra-Orthodox Jewish Men to Go to Work in Israel?. *Sociology of Religion, 85(4)*, 482-511.

Putnam, R. D. (2000). *Bowling alone: The collapse and revival of American community*. Simon & Schuster.

Rosen, B., Samuel, H., & Merkur, S. (2009). *Israel: Health system review*. Copenhagen: WHO Regional Office for Europe on behalf of the European Observatory on Health Systems and Policies.

Rosman, E. (2023). Haredim and Conscription to the IDF: Perspectives, Perceptions, Prospects. In K. Caplan & N. Leon (Eds.), *Contemporary Israeli Haredi Society* (pp. 90-109). Routledge.

Samuelson, W., & Zeckhauser, R. (1988). Status quo bias in decision-making. *Journal of Risk and Uncertainty, 1*(1), 7–59. https://doi.org/10.1007/BF00055564

Schatz, R. T., Staub, E., & Lavine, H. (1999). On the varieties of national attachment: Blind versus constructive patriotism. *Political Psychology, 20(1)*, 151-174.

Shalvi, S., Gino, F., Barkan, R., & Ayal, S. (2015). Self-serving justifications: Doing wrong and feeling moral*. Current Directions in Psychological Science, 24(2)*, 125–130. https://doi.org/10.1177/0963721414553264

Sorotzkin, D. (2022). The Formation of Ḥaredism—Perspectives on Religion, Social Disciplining and Secularization in Modern Judaism. *Religions, 13(2)*, 175.

Sosis, R., & Bressler, E. R. (2003). Cooperation and commune longevity: A test of the costly signaling theory of religion. *Cross-cultural Research, 37(2)*, 211-239.

Stadler, N. (2009). *Yeshiva fundamentalism: Piety, gender, and resistance in the ultra-Orthodox world.* New York University Press.

Stadler, N., & Ben-Ari, E. (2003). Other-Worldly Soldiers? Ultra-Orthodox Views of Military Service in Contemporary Israel. *Israel affairs, 9(4)*, 17-48.

Stadler, N., Lomsky-Feder, E., & Ben-Ari, E. (2008). Fundamentalism's encounters with citizenship: The Haredim in Israel. *Citizenship Studies, 12(3)*, 215-231.

Staetsky, L. D. (2022). *Haredi Jews around the world: Population trends and estimates*. Institute for Jewish Policy Research.

Suzin, A. (2025). Community Organization and Social Change: Examining Civil Society Development Across Israeli Ultra-Orthodox Groups. *Journal of Jewish Identities, 18(2)*, 287-306.

Tajfel, H., & Turner, J. C. (1986). The social identity theory of intergroup behaviour. In S. Worchel, & W. G. Austin (Eds), *Psychology of intergroup relations*. Chicago, IL: Nelson-Hall.

Tavris, C., & Aronson, E. (2007). *Mistakes were made (but not by me): Why we justify foolish beliefs, bad decisions, and hurtful acts.* Harcourt.

Thaler, R. H., & Sunstein, C. R. (2008). *Nudge: Improving decisions about health, wealth, and happiness*. Yale University Press.

Times of Israel. (2024, December 22). *Survey: Public support for conscripting ultra-Orthodox has surged throughout war.* Retrieved from https://www.timesofisrael.com/survey-public-support-for-conscripting-ultra-orthodox-has-surged-throughout-war/

Tsuria, R., & Campbell, H. A. (2021). “In My Own Opinion”: Negotiation of Rabbinical Authority Online in Responsa Within Kipa. co. il. *Journal of Communication Inquiry, 45(1)*, 65-84.

Zimmer, M., & Sellmann, M. (2024). The vitality of religious communities—a contribution model based on the sociological and theological discourse. *Zeitschrift für Religion, Gesellschaft und Politik, 8*(1), 81–107. https://doi.org/10.1007/s41682-023-00165-0

# Full Survey Questionnaire

Dear participant,

This questionnaire aims to understand the views and opinions of the ultra-Orthodox (Haredi) public in Israel regarding issues such as employment, military and national service, education, politics, and integration with the broader Israeli society. Our goal is to learn about existing attitudes and the degree of openness toward civic and social changes, alongside the preservation of a Haredi way of life. The survey is completely anonymous; no identifying information is collected. All data will be kept confidential and used only in aggregate form to contribute to both Haredi and general Israeli society. There are no right or wrong answers — we ask only for your honest responses. Participation is voluntary, and you may refuse or withdraw at any point without any consequences.

Estimated completion time: approximately 10 minutes.

Before we begin, please confirm your participation and indicate your preferred language form (masculine/feminine). The questionnaire version will adjust accordingly.

☐ I agree to participate and prefer the masculine form ☐ I agree to participate and prefer the feminine form ☐ I do not wish to participate (End Survey)

**1. How do you define yourself? (Screening question)**

a. Haredi (Ultra-Orthodox) b. Religious (non-Haredi) c. Traditional d. Secular e. Other

**Please indicate how much you agree with each statement (1 = Strongly disagree; 5 = Strongly agree):**

2. Haredim should be as economically independent as possible.

3. Acquiring a profession is important for Haredim.

4. Haredi participation in the general labor market should be encouraged.

5. It is appropriate to combine Torah study with gainful employment.

6. A Haredi who goes to work is considered “less Haredi.”

**7. What do you think is the main reason why Haredim pursue academic or professional studies? (Choose up to two reasons):**

a. Economic hardship or need to earn a living b. Personal interest in the field of study/work c. Desire to develop a career or fulfill personal potential d. Problems in the traditional educational framework e. Desire to integrate into broader Israeli society f. Other: ___________

**8. In your opinion, what is the best way to combine Torah study and livelihood?**

a. Part-time work alongside study b. Studying during the day and working in the evening c. Occupations specifically adapted for Haredim d. Gradual integration by age e. Other: ___________

**9. To what extent do you agree that core (general) studies should be included in Haredi educational institutions? (1–5)**

**10. Would you encourage your children or relatives to pursue academic studies?**

a. Yes, but only the sons b. Yes, but only the daughters c. Yes, both sons and daughters d. Neither sons nor daughters

**11. Who, in your opinion, should decide whether Haredim enlist in the military?**

a. The individuals themselves b. Haredi families c. The rabbis

**12. To what extent do you support a military service track adapted for Haredim?**

1. Strongly oppose

2. Moderately oppose

3. Slightly oppose

4. Unsure

5. Slightly support

6. Moderately support

7. Strongly support

**13. Every citizen, regardless of sector, should contribute to the state—whether through military or civilian service.**

(1 = Strongly disagree; 7 = Strongly agree)

**14. Would you recommend that a relative perform military or civilian service?**

a. Both military and civilian b. Civilian only c. Military only d. Neither

**15. What are the most important conditions for a Haredi military framework? (Select up to two)**

a. Strict observance of kosher laws b. A closed Haredi framework c. Integrated Torah study d. Haredi commanders e. A designated Haredi corps f. Adaptation of IDF values to Haredi values

**16. The principle of *Toratam Omanutam* (“their Torah is their vocation”) is no less important than the compulsory conscription law.**

(1 = Strongly disagree; 7 = Strongly agree)

**17. The Haredi community’s attitude toward military enlistment has changed in recent years.**

(1 = Strongly disagree; 5 = Strongly agree)

**18. Do you believe that Haredi representatives in government will succeed in passing a conscription law exempting Haredim from IDF service?**

a. Yes b. No c. Unsure

**19. If the conscription law fails to pass, do you think it will harm the power of Haredi parties?**

a. Definitely yes b. Probably yes c. Don’t know d. Probably not e. Definitely not

**20. In your opinion, does rabbinic leadership prevent reaching a compromise on the conscription issue?**

a. Yes b. No

**21. Where would you place yourself on the scale between conservative and modern?**

1. Very conservative

2. Conservative

3. In-between

4. Modern

5. Very modern

**22. How important is belonging to your community?**

a. Not important at all b. Slightly unimportant c. Quite important d. Very important

**23. In your view, is the Haredi society in Israel becoming more closed, more open, or remaining unchanged?**

a. Much more closed b. Somewhat more closed c. No change d. Somewhat more open e. Much more open

**24–29. To what extent do you consult the following people on major life decisions (family, education, political support)?**

(1 = Not at all; 5 = Very much)

24. A rabbi

25. A Rebbe (Admor)

26. A family member

27. A community friend

28. A professional

29. The media

**30. To what extent do you feel part of the State of Israel and its problems?**

a. Very much b. Quite a lot c. Moderately d. Slightly e. Very little

**31. I am proud to be Israeli.**

(1 = Strongly disagree; 5 = Strongly agree)

**32. Which party did you vote for in the last election?**

a. Shas b. United Torah Judaism c. Otzma Yehudit / Religious Zionism d. Likud

e. Other: __________ f. I did not vote

**33. How satisfied are you with the party you voted for?**

a. Very satisfied b. Satisfied c. Somewhat dissatisfied d. Not satisfied e. Disappointed

**34–37. Please indicate how much you agree with the following statements (1 = Strongly disagree; 5 = Strongly agree):**

34. The Haredi parties represent my needs properly.

35. I would never vote against my rabbi’s opinion.

36. It is important to allow more personal choice within the Haredi public.

37. The Haredi community rejects those who vote differently from the majority.

**38. There is room for a new Haredi–national party.**

(1 = Strongly disagree; 5 = Strongly agree)

**39. If a liberal Haredi party promoting employment, education, conscription, and equality were established, would you consider voting for it?**

a. Definitely yes b. Probably yes

c. Undecided d. Probably not e. Definitely not

**40. If the same party focused only on employment, education, and equality—but not on conscription—would that affect your view?**

a. Definitely yes b. Probably yes c. Not sure d. Probably not e. Definitely not

**41. How many of your Haredi friends would consider voting for such a party?**

a. None b. A small minority c. A considerable number d. Most of them e. Almost all

**42. Which factors might influence your willingness to vote for such a party?**

a. If it were led by a God-fearing Haredi b. If it pledged to uphold Torah values c. If it had rabbinic legitimacy d. If it promoted Haredi family welfare

e. If other Haredim supported it f. Other: ___________

**43. What are the main obstacles to establishing such a party? (Select all that apply)**

a. Lack of rabbinic support b. Pressure from within the community c. Fear of losing Haredi identity d. Distrust of politicians e. Lack of awareness of the option f. Other: ___________

**44. Gender**

a. Male b. Female

**45. Age: _______**

**46. Marital status**

a. Single b. Married c. Widowed d. Divorced

**47. Number of children: _______**

**48. Place of residence: ___________**

**49. Which Haredi subgroup do you belong to?**

a. Lithuanian (Litvish) b. Hasidic c. Sephardic Haredi d. Modern Haredi e. Chabad f. Breslov g. General Haredi h. Eda Haredit i. Unaffiliated Haredi j. Ba’al Teshuva (returnee to religion) k. Other: ___________

**50. What is your highest educational qualification?**

a. No formal certificate b. External exams (seminary-level) c. Full matriculation certificate d. Post-secondary non-academic program e. Bachelor’s degree f. Master’s degree g. Doctorate h. Other: ___________

**51. Current main occupation**

a. Full-time Torah scholar (Kollel) b. Yeshiva student c. Religious worker (Torah-related) d. Non-religious occupation e. Student (academic or vocational) f. Unemployed – seeking work g. Retired/pensioner h. Homemaker i. Other: ___________

**52. Monthly household income**

a. No income b. Up to 5,000 NIS c. 5,000–10,000 NIS d. 10,000–15,000 NIS e. 15,000–25,000 NIS f. Above 25,000 NIS

**53. Do you own a mobile phone?**

a. None b. Kosher non-smartphone c. Kosher smartphone d. Non-kosher smartphone e. Both kosher and non-kosher phones

**54. Do you have Internet access at home?**

a. No b. Yes, filtered (“Etrog” / “Netiv”) c. Yes, lightly filtered (“Rimon” etc.) d. Yes, unrestricted Internet

**55. Which newspapers or magazines are regularly present in your home?**

a. None b. Only Haredi newspapers/magazines c. Only Haredi children/youth magazines d. Both Haredi and general newspapers e. Other: ___________

**56. Which radio stations do you usually listen to (other than news)?**

a. Do not listen / only to news b. Only Haredi radio stations c. Both Haredi and general stations d. Only general stations

**57. Is there anything else you would like to share regarding the topics discussed in this questionnaire?**

_________________________________________________________________________

**Thank you very much for your participation!** Your responses are valuable for understanding the ongoing changes within Israel’s Haredi society. For further information, please contact: **Prof. Guy Hochman – ghochman@runi.ac.il**
